# Safety and efficacy of 2% chlorhexidine gluconate aqueous versus 2% chlorhexidine gluconate in 70% isopropyl alcohol for skin disinfection prior to percutaneous central venous catheter insertion in preterm neonates: the ARCTIC randomised-controlled feasibility trial protocol

**DOI:** 10.1136/bmjopen-2018-028022

**Published:** 2019-02-08

**Authors:** Paul Clarke, Jean V Craig, John Wain, Catherine Tremlett, Louise Linsell, Ursula Bowler, Ed Juszczak, Paul T Heath

**Affiliations:** 1 Neonatal Unit, Norfolk and Norwich University Hospitals NHS Foundation Trust, Norwich, Norfolk, UK; 2 Norwich Medical School, University of East Anglia, Norwich, UK; 3 Research Design Service, Norwich Medical School, University of East Anglia, Norwich, UK; 4 Quadram Institute Bioscience, Norwich Research Park, Norwich, UK; 5 Department of Microbiology, Norfolk and Norwich University Hospitals NHS Foundation Trust, Norwich, UK; 6 National Perinatal Epidemiology Unit, Nuffield Department of Population Health, University of Oxford, Oxford, UK; 7 Paediatric Infectious Diseases Research Group, Infection and Immunity, St George’s University of London, London, UK

**Keywords:** antiseptic, biocide, disinfection, central Line associated bloodstream infection, catheter

## Abstract

**Introduction:**

Catheter-related sepsis is one of the most dangerous complications of neonatal intensive care and is associated with significant morbidity and mortality. Use of catheter-care ‘bundles’ has reduced the incidence of catheter-related sepsis, although individual components have not been well studied. Better evidence is needed to guide selection of the most appropriate antiseptic solution for skin disinfection in preterm neonates. This study will inform the feasibility and design of the first randomised controlled trial to examine the safety and efficacy of alcohol-based versus aqueous-based chlorhexidine antiseptic formulations for skin disinfection prior to percutaneous central venous catheterisation in preterm neonates. The antiseptics to be compared are 2% chlorhexidine gluconate (CHG) aqueous and 2% CHG in 70% isopropyl alcohol.

**Methods and analysis:**

The Antiseptic Randomised Controlled Trial for Insertion of Catheters (ARCTIC) is a two-centre randomised-controlled feasibility trial. At least 100 preterm infants born at <34 weeks’ gestation and due to undergo percutaneous insertion of a central venous catheter will be randomly allocated to receive prior skin disinfection with one of the two antiseptic solutions. Outcomes include: i) recruitment and retention rates; ii) completeness of data collection; iii) numbers of enrolled infants meeting case definitions for definite catheter-related sepsis, catheter-associated sepsis and catheter colonisation and iv) safety outcomes of skin morbidity scores recorded daily from catheter insertion until 48 hours post removal. The key feasibility metrics will be reported as proportions with 95% CIs. Estimated prevalence of catheter colonisation will allow calculation of sample size for the large-scale trial. The data will inform whether it will be feasible to progress to a large-scale trial.

**Ethics and dissemination:**

ARCTIC has been approved by the National Health Service Health Research Authority National Research Ethics Service Committee East of England (Cambridge South) (IRAS ID 163868), was adopted onto the National Institute of Health Research Clinical Research Network portfolio (CPMS ID 19899) and is registered with an International Standard Randomised Control Trials Number (ISRCTN82571474; Pre-results) and European Clinical Trials Database number 2015-000874-36. Dissemination plans include presentations at scientific conferences, scientific publications and sharing of the findings with parents via the support of Bliss baby charity.

**Trial registration number:**

ISRCTN82571474; Pre-results.

**Strengths and limitations of this study**

- The Antiseptic Randomised Controlled Trial for Insertion of Catheters study will be one of only very few randomised controlled trials of skin antiseptics in preterm neonates and the first to compare aqueous 2% chlorhexidine gluconate versus 70% isopropyl alcohol-based 2% chlorhexidine gluconate for cutaneous disinfection prior to central venous catheterisation.
- The trial will collect rigorous, prospective safety data following antiseptic application through daily skin safety assessments using a validated neonatal skin scoring tool.
- This will be the first study in neonates to undertake molecular typing of isolates to verify that skin-colonising and blood-cultured organisms match catheter-colonising organisms to a species level in babies with suspected sepsis, thus allowing definitive proof of catheter-related sepsis.
- Catheter colonisation will be used as a proxy for catheter sepsis, and the target sample size is based on an anticipated incidence of catheter colonisation of 20% in the reference antiseptic group, estimated with a 95% CI of 11% to 31%; the study is not powered to detect differences in clinical outcomes.
- This trial will show whether a future large-scale multicentre randomised controlled non-inferiority trial of the same antiseptics is feasible and will determine the sample size required for such a trial.

## Introduction

Percutaneously inserted central venous catheters (PCVCs) are inserted daily in neonatal intensive care units (NICUs) across the world to deliver hyperosmolar parenteral nutrition solutions to preterm neonates. PCVCs may remain in situ for weeks,^1^ but their presence entails a major risk for bloodstream infection. In a previous study, 32% of inserted PCVCs were colonised with potentially pathogenic bacteria at the point of removal, and 8% overall were associated with definite catheter-related sepsis (CRS).^1^ Extraluminal colonisation is the main route of catheter colonisation in short-term CVCs: skin bacteria traverse the catheter insertion site onto the catheter, colonise the line and act as focus for CRS.^2^ In one study, the presence of skin bacteria at the catheter exit site was associated with an 8-fold increased risk of catheter colonisation and a 10-fold increased risk of CRS caused by the same organism.^2^

For preterm babies in the NICU, CRS is a dangerous complication associated with significant morbidity and mortality. Sepsis increases the duration of intensive care and hospitalisation, need for antibiotics and risks for adverse neurodevelopmental outcome. Coagulase-negative staphylococcal infections cause the majority of CRS in the NICU (80%–90%), may be life-threatening and can cause permanent lifelong injury and disability in survivors, including cerebral palsy.^3 4^

Reduction of CRS has been a major goal of the National Health Service for the past decade.^5^ Catheter-care ‘bundles’, guidelines incorporating collected good practices for catheter insertion and maintenance, have successfully reduced the incidence of catheter colonisation and CRS in the NICU,^6^ although await universal adoption.^7^

The individual components of catheter-care bundles have not been well studied in randomised controlled trials (RCTs). Adequate skin disinfection of the catheter insertion site is arguably the most important component of catheter-care bundles to prevent catheter colonisation and CRS. Optimal skin preparation will abolish or significantly reduce numbers of skin organisms, so limiting risks of residual skin colonisation by bacteria that may then colonise a PCVC and cause CRS.

Studies in adults, including meta-analysis, show that alcohol-based antiseptics are superior for topical antisepsis,^8 9^ and UK national evidence-based guidelines recommend use of 2% chlorhexidine gluconate in 70% isopropyl alcohol (2%CHG-70%IPA) for skin antisepsis in adults and older children. However, there is no guidance for preferred antiseptic in infants, including preterm infants, due to the lack of evidence and specific safety concerns in this population.^10 11^ The best antiseptic to use for preterm babies is still unknown, and multiple different antiseptics, combinations and concentrations are presently being used in UK NICUs; approximately half use a 2% concentration of CHG and 60% an IPA-containing CHG formulation.^12^ For preterm neonates, there is no Cochrane review comparing skin antiseptics for cutaneous disinfection prior to PCVC insertion, and only two RCTs have compared topical antiseptics for PCVC insertion.^13 14^

There are risks associated with antiseptic use peculiar to preterm infants. Their thin skin is vulnerable to chemical injury and absorption. Chemical skin burns have been described with all the currently used topical antiseptics, including both aqueous and alcohol-containing CHG formulations, and iodine solutions,^15^ as well as with octenidine.^16^ Topical alcohol use may also increase the risk of systemic chemical absorption.^17^

There are no published RCTs that have examined the safety and efficacy of alcohol-based versus aqueous CHG formulations for neonatal antisepsis. This feasibility study aims to inform the safety and assist the design and planning of a future large-scale multicentre RCT that will examine whether 2% CHG aqueous is non-inferior in antiseptic efficacy compared with 2%CHG-70%IPA for skin disinfection prior to PCVC insertion in preterm neonates. An aqueous CHG is likely to have fewer side effects than an alcohol-based CHG, and would therefore be preferable if found to be non-inferior in terms of antisepsis.

## Objectives

To determine the proportion of babies in the 2%CHG-70%IPA group with colonisation of at least one of the two catheter segments taken at catheter removal. Catheter colonisation will be the primary outcome in the full-scale trial because it is a valid surrogate for CRS^2^; to determine factors affecting recruitment and process outcomes that will help refine the design of the large-scale trial; also to estimate numbers of enrolled infants who have definite CRS and numbers with catheter-associated sepsis, determine suitability and completeness of data collection methods and describe any skin morbidity occurring in trial participants related to use of the study antiseptics.

## Methods and analysis

### Study design

A feasibility, masked RCT of Investigational Medicinal Products (IMPs). Preterm infants born at <34 weeks’ gestation who are due to undergo planned insertion of a PCVC will be randomised to receive one of two topical disinfection agents for skin antisepsis: 2% chlorhexidine gluconate aqueous (2%CHG-aqueous) or 2%CHG-70%IPA.

#### Study setting

Two tertiary-level neonatal units in the UK, Norfolk and Norwich University Hospital and Medway Maritime Hospital, which each cater to a total of 5000–6000 deliveries per year.

## Eligibility

### Inclusion criteria

- Preterm infants born at <34 weeks’ gestation.
- Requiring routine insertion of a PCVC for parenteral nutrition.
- No new episode of suspected sepsis with commencement of antibiotics occurring within the 48 hours preceding planned catheter insertion.
- No other indwelling PCVC already in situ.

### Exclusion criteria

- No realistic prospect of survival in the short term.
- Life-threatening congenital abnormality.
- Underlying skin condition.
- Another indwelling PCVC already in situ or previously enrolled into the study.
- Positive blood culture (BC) within the past 7 days without a subsequent negative BC result.
- Antibiotic treatment commenced for suspected sepsis within the preceding 48 hours.

### Key definitions

*Definite* CRS: a peripheral BC plus any catheter segment (ie, one of the ~1 cm long proximal or tip catheter portions) positive with the same organism, based on bacterial culture, antibiotic sensitivity and molecular typing, from a neonate who had an indwelling PCVC and clinical signs of sepsis but no other focus of sepsis.

*Catheter colonisation*: a catheter that at the time of removal has either one or both segments that are culture positive.

*Catheter-associated sepsis*: a baby with clinical signs of sepsis and an accompanying positive BC in the period between catheter insertion and 48 hours post removal but who has no other focus of sepsis and in whom both catheter segment cultures are negative.

### Recruitment

Preterm babies potentially suitable for the trial will be identified by the clinical healthcare teams. Parents of such infants will be approached for consent by the research team or delegated suitably qualified member of the clinical healthcare team trained in study procedures and Good Clinical Practice (GCP). A written parent information sheet that forms part of the Parental Informed Consent Form will be provided to help explain the study (online supplementary file 1). Written maternal consent will be obtained and countersigned by the person who obtained informed consent (principal investigator (PI), or appropriately qualified healthcare professional with delegated authority).

10.1136/bmjopen-2018-028022.supp1 Supplementary file 1

### Randomisation

Following consent, randomisation to either 2%CHG-70%IPA antiseptic or 2%CHG-aqeuous antiseptic will take place as close as possible to the time of planned catheter insertion. Randomisation will be managed via a secure web-based facility hosted by the National Perinatal Epidemiology Unit Clinical Trials Unit (CTU) and will use a 3:1 allocation ratio in favour of the 2%CHG-70%IPA antiseptic group, the group that will inform the power calculation for the large-scale trial. Groups will be stratified by birth gestation (<28 weeks; 28 weeks+0 days to 33 weeks+6 days) and by centre. Treatment allocation will be masked such that the allocation will not be known by clinicians, the baby’s family, laboratory staff or trial outcome assessors.

### Interventions

**Figure 1:**
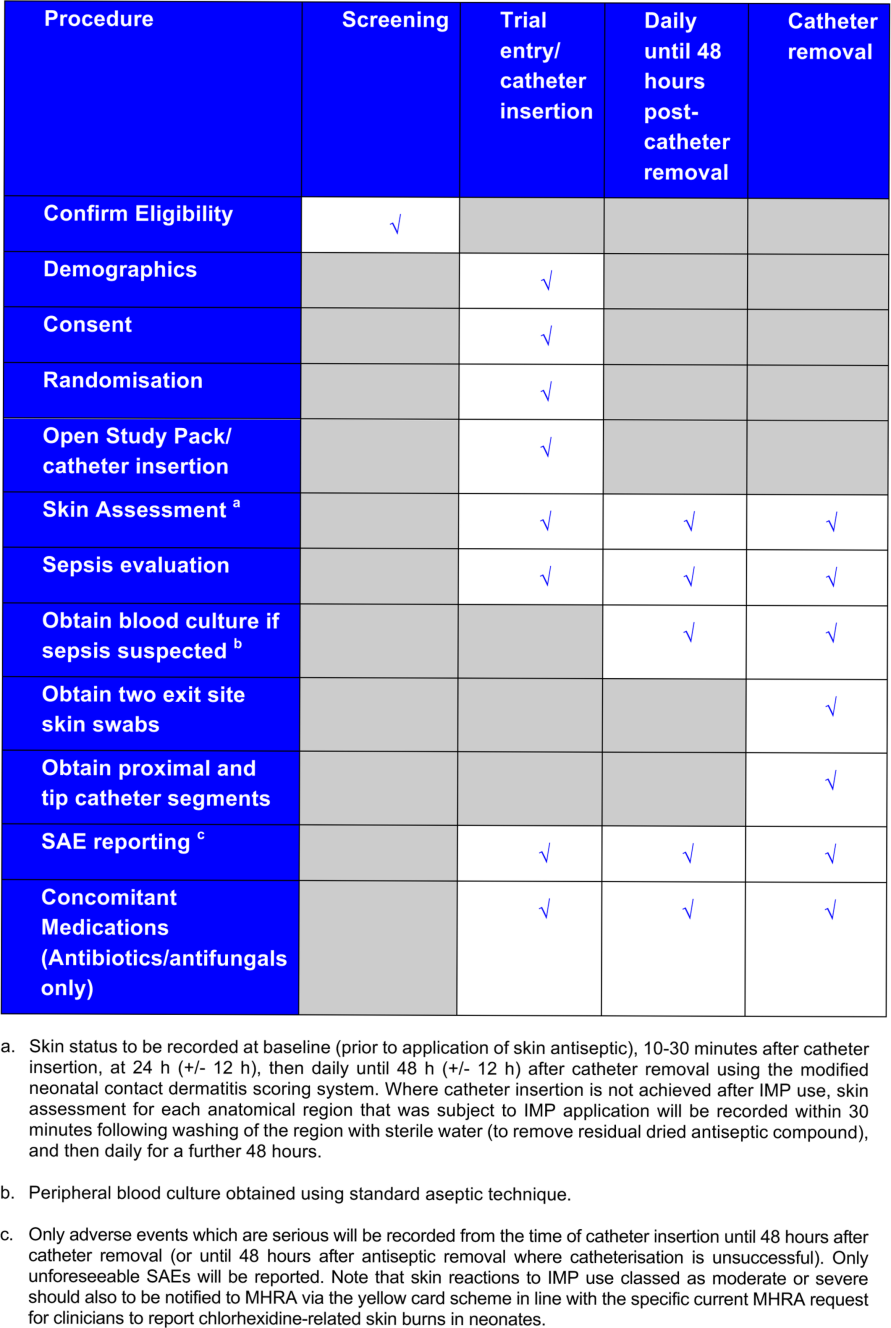
Trial procedures.

The trial procedures are summarised in figure 1.

### Skin disinfection at catheter insertion

Study packs stored in a locked, secure, temperature-monitored cupboard will contain two bottles of the same allocated antiseptic Investigational Medicinal Product (IMP) each labelled with the same identifying code. One bottle will be opened at catheter insertion, the other will be retained for use at catheter removal. The disinfection procedure requires sparing application of allocated antiseptic solution for 10–20 s from sterile gauze to the skin site area selected for catheterisation. Instructions are: use only the minimal volume of antiseptic necessary for skin coverage, avoid any pooling of antiseptic, ensure that any excess solution and any soaked drapes or gowns are removed to avoid any prolonged contact with the skin and allow the disinfected area to air dry completely (for ≥30 s) before proceeding with catheter insertion. Excepting in the case of failed catheterisation, saline or water must not be used to wipe the disinfected skin area following application of antiseptic solution.

### Catheter insertion

Catheter insertion will be standardised, using a working guideline common to both participating centres that requires strict aseptic technique and encompasses established good clinical practices for PCVC insertion and care adopted from catheter-care bundles^6 8 18 19^ The decision to insert a PCVC and choice of catheter (Premicath 1Fr/28G or Epicutaneo-Cava catheter 2Fr/24G: both Vygon (UK), Cirencester, UK) is at the discretion of the attending clinical team. All personnel involved will be trained in catheter insertion and maintenance procedures. An insertion checklist will be completed for all catheterisations and a disposable face mask will be worn by the operator inserting the catheter for asepsis purposes and also to minimise the risk of possible unblinding from any smell of alcohol.

### Assessment of skin condition

Skin status will be recorded using a validated neonatal contact dermatitis scoring system, the Neonatal Skin Condition Score,^20^ with minor modification. Assessments will be undertaken by a nurse trained in use of the scoring system and will be recorded at baseline (prior to application of antiseptic), within 10–30 min after catheter insertion and then daily until 48 hours following catheter removal, or daily for 48 hours after antiseptic application in cases where catheterisation is unsuccessful. Serious chemical skin burns adjudged by a PI or delegate to be severe or moderately severe, will be notified to the Medicines and Healthcare Products Regulatory Agency (MHRA).^21^

### Catheter removal and obtainment of study specimens

Catheters are usually removed when no longer required, although sometimes removal is warranted earlier than intended because of complications, including suspected sepsis. If a catheter is being removed from a baby with suspected sepsis then a concurrent peripheral BC will be obtained as per routine clinical practice. The decision for catheter removal for enrolled babies lies with the attendant clinical team.

The following samples will be obtained at the time of catheter removal for microbiological analysis:

- Two exit site skin swabs taken *before* catheter removal: the first after removing all covering dressings but prior to skin disinfection; the second taken post new disinfection of the insertion site, once the antiseptic has dried. This disinfection procedure is intended to limit the risk of catheter contamination by residual skin organisms during the removal of the catheter and will use the same allocated antiseptic as was used for catheter insertion. Both specimens will be obtained by rolling the swab tip several times across the skin of the catheter insertion site, over an area within <0.5 cm radius of the insertion site and including the actual puncture site.
- Two approximately 1 cm long catheter segments (proximal and tip) *after* catheter removal. Proximal catheter segments have higher colonisation rates than tips,^1^ therefore microbiological analysis of both catheter segments rather than the tip alone may improve the diagnostic yield of catheter colonisation.

**Figure 2:**
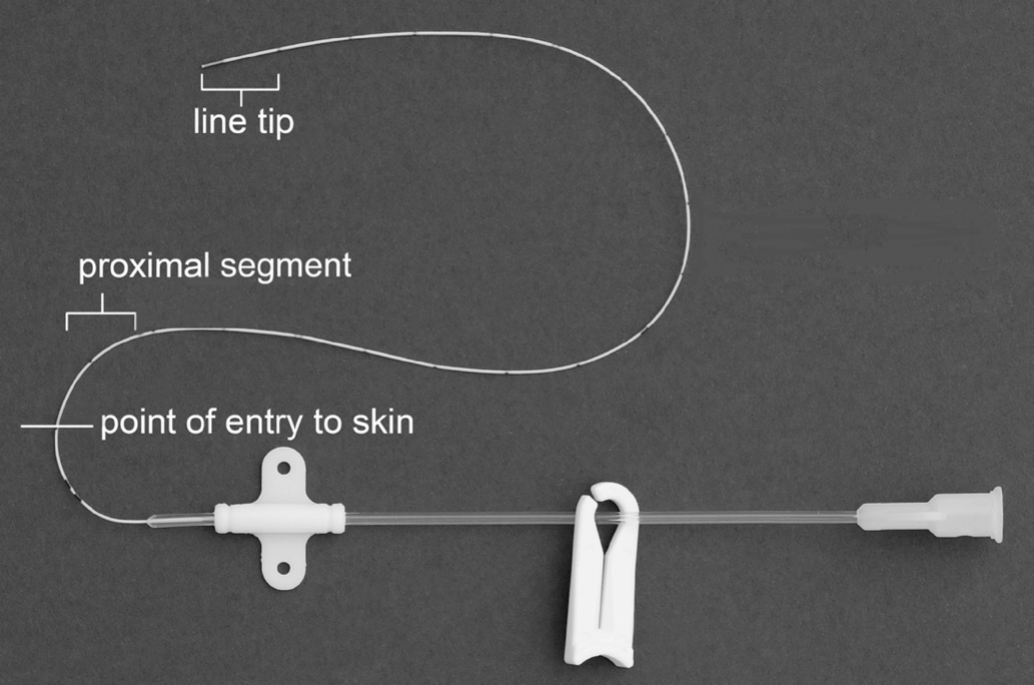
Picture showing catheter sections taken at catheter removal.

Catheter removal requires two trained persons and care to avoid cross-contamination between segments while sectioning the catheter. Two separate sets of sterile forceps, two pairs of sterile scissors and two sterile prelabelled universal containers are required. Before removing the catheter, the subcutaneous insertion length will be noted from the external catheter markings. The catheter will be removed onto a sterile paper towel field then sectioned using separate pairs of sterile scissors to obtain two ~1 cm long formerly subcutaneous catheter segments: i) tip and ii) a proximal segment, taken approximately 1–2 cm distal to the point of skin entry (figure 2). The individual segments will be placed into separate appropriately labelled sterile universal containers using separate pairs of sterile forceps.

### Microbiology

The catheter segments and skin swabs will be submitted to the local microbiology laboratories for routine culture and antibiotic sensitivities. BCs sent from babies with suspected sepsis at the time of catheter removal will undergo standard culture methods. Bacterial growths from skin swab cultures will be assessed using a semi-quantitative method.^22^ All laboratory staff will be blinded to antiseptic allocation. Isolates from culture-positive skin swabs, blood and catheter segmental cultures will be retained for molecular typing. Initial identification of organisms will be done by mass spectrometry. Those giving similar patterns will be analysed using next-generation sequencing using a multiplexed approach on the Illumina MiSeq. Molecular typing of paired blood and catheter isolates from the same baby will allow confirmation that isolates are identical to a species level, for definitive diagnosis of definite CRS. While few postdisinfection skin swabs are expected to be positive, molecular typing of skin swab isolates will be done for any babies with colonised catheters: isolation of paired identical species could indicate possible catheter contamination by skin organisms during catheter removal. Babies with positive BCs will be managed according to local clinical guidelines; involvement in this trial will not dictate or influence clinical antibiotic prescriptions.

### Outcome measures

- Proportion of babies in the 2%CHG-70%IPA arm with catheter colonisation as determined by positive bacterial culture from one or both of the catheter segments taken at catheter removal (primary outcome).
- Rates of recruitment and retention to the study, and the collection of views of parents and clinicians on factors affecting recruitment and retention.
- Proportion of infants with positive exit-site skin swabs at catheter removal.
- Number and type of catheter segments culture positive at removal.
- Bacterial species (typed via molecular methods) of isolates identified on positive BC, exit-site skin swabs and catheter segments.
- Proportion of infants undergoing an infection screen in the period between catheter insertion and 48 hours post catheter removal that meets case definition for definite CRS.
- Proportion of infants with positive blood culture from any infection screen in the period between catheter insertion and 48 hours post catheter removal that meets definition for catheter-associated sepsis.
- Rate of CRS per 1000 PCVC days.
- Rate of catheter-associated sepsis per 1000 PCVC days.
- Proportion of infants completing the study with complete data for the primary outcome and proportions of infants with missing data collection forms.
- Daily skin morbidity scores in the period between catheter insertion and 48 hours post catheter removal, and in the period between antiseptic application and 48 hours post antiseptic application where catheterisation was unsuccessful.

### Supportive care of participants

The clinical management of babies enrolled in the study will follow standard local practices. For the purposes of this feasibility study, if CRS is suspected the ideal will be catheter removal at that time. However, it is recognised that a pragmatic approach is sometimes needed, especially for very premature babies in whom catheter replacement may be difficult and challenging. Efforts will be made to minimise local differences in treatment practices between sites through training.

### Discontinuation of trial intervention

The trial intervention will be stopped on parental request, or if the baby develops serious adverse skin damage that, in the opinion of the responsible PI, was caused by the IMP. Thus, if any baby has a clinically significant chemical skin burn following IMP application at catheter insertion then the allocated antiseptic will be withheld from use for skin disinfection at catheter removal. In such instance, skin swab and catheter sections will still be obtained as per removal procedure and the protocol deviation will be recorded.

### Blinding and unblinding

The antiseptic IMPs will be manufactured by a MHRA-accredited Specialist Pharmacy Manufacturing Unit compliant with Good Manufacturing Practices. The IMP will be supplied in bottles and both products will be coloured pink (using carmoisine) and visually indistinguishable. To maintain blinding, each baby will be issued a unique allocation number corresponding to the study pack number. Emergency unblinding for valid medical or safety reasons is via the randomisation website using a single-use access code provided in a sealed envelope in the Investigator Site File.

### Sample size

Our previous study found that 32% of PCVCs had a colonised tip and/or proximal segment at removal after using much weaker concentration (0.015%–0.05%) CHG solutions and the definite CRS rate was 6.8 per 1000 catheter days.^1^ In comparison, a UK NICU that routinely used 2%CHG-70%IPA for PCVC insertions reported a 34% lower CRS rate (4.5 per 1000 catheter days).^18^ Presuming that alcohol-based 2%CHG is the major factor of benefit in catheter care, by extrapolation we might reasonably expect to see a 34% reduction in extraluminal catheter colonisation rates by using 2%CHG-70%IPA solution. Thus, we estimate a catheter colonisation rate of approximately 20% (0.66×32%=21%) may be achieved with 2%CHG-70%IPA solution. A 3:1 allocation ratio in favour of the reference 2%CHG-70%IPA group requires a target sample size of approximately 93 babies with successfully inserted (and removed) catheters (ie, n=70 in the reference group) to estimate the critical parameters for a future, large-scale trial with an adequate degree of precision. With this target sample size, the anticipated incidence of the primary outcome in the reference group of 20% will be estimated with a 95% CI of 11% to 31%. With a sample size of 93 babies with successfully inserted catheters, the anticipated recruitment/uptake rate of 75%^13^ will be estimated with a 95% CI of 0.65 to 0.83. A sample size of approximately 93 babies having catheters successfully inserted/removed will require parents of at least 124 eligible babies to be approached.

### Data management and analysis

Outcome data include routinely recorded clinical information obtainable from clinical and local microbiological laboratory records. Data verifying species of catheter colonisation will be collected following further analysis of positive isolates by molecular typing. Data will be collected using study-specific data collection forms for: trial entry and randomisation, main outcome data (catheter insertion, skin condition assessment, sepsis evaluation and antimicrobial therapy), catheter removal, microbiology data, unsuccessful catheterisation episodes, discontinuation of intervention, withdrawal and foreseeable serious adverse events (SAEs). In addition, information will be collected and reported to the sponsor using the sponsor’s SAE report form and incident form, to report any deviation from the protocol, trial-specific procedures or GCP.

Data collection will proceed from randomisation until 48 hours post catheterisation for successfully inserted catheters, or until 48 hours after last antiseptic application for unsuccessful catheterisation. If a baby is discharged from its recruiting centre before study completion, to try to achieve complete follow-up safety data, the research team will contact the receiving clinical nursing team to request routine daily documentation regarding status of catheter insertion site skin and details of any new clinical sepsis events until 48 hours post catheter removal.

All data will be collected, transferred and stored in compliance with GCP and current data protection legislation. The trial co-ordinating centre (Norwich) will hold the main administrative database for the trial. Data acquired by the enrolling units will initially be recorded onto paper data collection forms, followed by entry into an OpenClinica database (OpenClinica, Waltham, USA) administered by the National Perinatal Epidemiology Unit. Access to this database will be via a web browser and restricted to authorised users. The database has been tested and validated prior to use. All data collection, transfer and storage will comply with GCP and Data Protection legislation.

### Statistical analysis plan

A statistical analysis plan for proposed analysis and presentation of the results of the trial will be drawn up by the delegated CTU medical statistician. Drafts will be reviewed by CTU personnel, by the CI and by the chair of the Trial Steering Committee (TSC) and a final version will be approved prior to the end of recruitment. Any deviations from the plan will be described and justified in the final report. Analysis will be carried out by an appropriately qualified and experienced statistician, who will ensure the integrity of the data during their processing.

### Site training

Each recruiting centre is staffed by a local research nurse dedicated to support the study. Initiation visits at each participating neonatal unit will be performed by the CI and study research nurse, also attended by the sponsor’s representative. Training in study-specific procedures and in awareness of the principles of GCP will be provided for nursing and medical staff in each site by the local PI and research nurses, who will also help maintain training and delegation logs.

### Monitoring

The sponsor’s nominated representatives will undertake monitoring visits during the course of the study at each recruiting site to check for completeness and quality of data collection and adherence to the study protocol and reporting requirements. A monitoring plan is in place to determine the frequency and scope of site monitoring based on continuing risk review. Face-to-face monitoring visits will initially be undertaken within the first 6 months and the frequency and mode of ongoing site monitoring will be revised following assessment of recruitment rates, number of data queries and safety/incident reports.

### Pharmacovigilance

Safety of participants will be assessed continuously from randomisation until 48 hours post catheter removal. The frequency of adverse events and SAEs as defined by The International Council for Harmonisation and that would normally require reporting within a clinical trial is expected to be high in this population. In accordance with regulatory guidance which allows for exceptions in such circumstances, a modified reporting plan was approved by the research ethics committee and by the MHRA. This plan exempted the need for routine reporting of prespecified SAEs that are a foreseeable occurrence in preterm babies, unless considered causally related to IMP or trial procedures. Unforeseeable SAEs will be reported on the sponsor’s SAE form. The relationship of each adverse event to the trial medication will be determined by a medically qualified individual. All reportable SAEs with causality assessed as ‘possibly’, ‘probably’ or ‘definitely’ will be considered as related to IMP. All SAEs assigned by the PI or delegate (or following sponsor/CI review) as suspected to be related to IMP *and* unexpected will be classified as suspected unexpected serious adverse reactions and subject to expedited reporting to the MHRA.

### Data and safety monitoring

The Data Monitoring Committee (DMC) is responsible for safeguarding the interests of the trial participants and making recommendations to the TSC. The ARCTIC DMC roles, responsibilities and operating procedures are defined in the ARCTIC DMC Charter. The DMC is composed of three independent multidisciplinary experts who are not involved in the conduct of the trial in any way. They met prior to the initiation of enrolment and determined a plan to review the protocol, compliance, safety and outcome data after 50 babies had been recruited. The TSC is composed of eight independent members and has a Charter defining members’ roles and responsibilities. Its Chair and the majority of the TSC membership are independent of the trial. The TSC provides the overall supervision, monitors progress and conduct of the trial and advises on its scientific credibility. The TSC will consider and act, as appropriate, on any recommendations of the DMC and carries ultimate responsibility for deciding whether the trial needs to be stopped on grounds of safety or efficacy. The TSC will report on trial progress to the trial funder.

### Patient and public involvement

The study proposal benefited from extensive patient and public involvement during its development. Advice received included regarding content of the Parental Informed Consent Form and aspects of protocol design. Input was received from Bliss baby charity (www.bliss.org.uk), by PPIRes (http://nspccro.nihr.ac.uk/public-and-patient-involvement-in-research) and by a consumer member representative of the Neonatal Clinical Specialty Group of the Medicines for Children Research Network. We also consulted with a local parent support group and with parents of babies who had suffered CRS. Two lay members are involved in trial management as members of the TSC, and will be involved in dissemination of findings.

### Ethics and dissemination

Clinical trial authorisation was granted by the MHRA (REF: 13630/0009/001–0001). Written approvals were received from individual hospital sites prior to recruitment. The trial commenced recruitment under protocol V.3.0, dated 18 November 2016; the full protocol is available at: https://www.npeu.ox.ac.uk/arctic. The investigator or a suitably qualified person designated by the local PI will obtain written informed consent from the patient’s parent/legally accepted representative before any trial-specific activity is performed. The CI will ensure that the trial is conducted in accordance with the principles of the Declaration of Helsinki and in conformity with GCP. The trial’s findings will be presented at national and international scientific meetings and conferences and will be published in an open access peer-reviewed journal.

## Conclusions

Recruitment to ARCTIC commenced in March 2017 and the projected overall trial end date is 31 March 2019. It is hoped that the findings of this feasibility study will pave the way for the definitive large-scale efficacy/safety study. The anticipated large-scale study will be a multicentre non-inferiority RCT of the same two antiseptics for skin disinfection prior to PCVC insertion in preterm neonates. Primary outcome will be catheter colonisation as determined by culture of one or more catheter segments taken at catheter removal. National evidence-based guidelines for preventing healthcare-associated infections in the NHS (‘epic’), commissioned by the Department of Health, were published in 2001, 2007 and 2014 to incorporate new research evidence.^10 11^ Due to the lack of previous quality RCTs in the preterm population, no previous guidelines included advice or recommendations on antiseptics specific for preterm neonates. We anticipate that the findings from this research will be incorporated into a future version of the epic guidelines.

## Supplementary Material

Reviewer comments

Author's manuscript
