# Supplementary material for: Safety and efficacy of 2% chlorhexidine gluconate aqueous versus 2% chlorhexidine gluconate in 70% isopropyl alcohol for skin disinfection prior to percutaneous central venous catheter insertion in preterm neonates: the ARCTIC randomised-controlled feasibility trial protocol
Source: BMJ Open. 2019 Feb 8;9(2):e028022. doi: 10.1136/bmjopen-2018-028022 (PMC6411256; doi:10.1136/bmjopen-2018-028022)
Supplement: Supplementary file 1 [file bmjopen-2018-028022supp001.pdf]

(*to be presented on NHS Trust headed paper*)

ARCTIC
Antiseptic Randomised Controlled Trial
for Insertion of Catheters

INFORMATION FOR PARENTS ABOUT A RESEARCH STUDY

Antiseptic Randomised Controlled Trial for Insertion of Catheters – The ARCTIC Study –

Dear Parents,

We would like to invite you to have your baby take part in a research study that is comparing two different skin antiseptics for cleaning the skin of babies. This leaflet explains the purpose of our study and what would be involved if you choose to allow your baby to take part.

Why am I being asked about this study now?
We are approaching you now because your baby will soon need to have a special feeding line inserted as part of their routine treatment on our neonatal unit. The special feeding line is a central venous catheter (CVC). This CVC is often also referred to as a 'long line' because it is a long, very thin silicone tube that is placed into one of your baby's arm/leg veins like a drip. It is 'long' because we aim to place the end of it in one of the big veins that drain into the heart. The CVC is needed to provide liquid nutrition that will help your baby to grow properly and become stronger. The CVC will stay in place until your baby manages to take all the milk needed for growth by mouth or feeding tube. These CVCs are usually required for at least 1-2 weeks, though sometimes for longer.

The study we are doing is looking at what antiseptic may be better to use to clean a baby's skin at the time when a CVC is inserted. We would therefore like you to consider allowing your baby to join this study before they have their CVC inserted in the next couple of days.

Are any problems possible with central venous catheters?
Insertion of CVCs into babies born prematurely is a routine procedure in neonatal units across the world. While these catheters are essential for delivering liquid nutrition and medicines to babies, one problem with their use is that they can sometimes attract bacteria and so lead to a bloodstream infection. Infection can be dangerous for small babies and so if there are concerns about possible infection in a baby who has a CVC in place, we would usually remove the catheter and treat the baby with antibiotics.

How can catheter infection be prevented?
A lot of research has already been done to understand and prevent catheter infections. Various good practices have been identified for insertion and management of CVCs, and you may be reassured to hear that our unit has adopted the good practices.

Why is this study being done?
One important aspect of catheter care to minimise infection is good skin disinfection at the time a CVC is inserted. Skin germs are the commonest cause of bloodstream infections in premature babies so choosing an effective skin antiseptic is clearly very important. Yet little research has been done in this area in babies, so we are trying to find out what antiseptics are best to use for CVC insertion in premature babies. Good antiseptic skin preparation before CVC insertion can significantly reduce the number of germs present on the skin and so reduce the risk of the skin germs colonising (growing onto) the catheter and leading to catheter infection. While it is standard practice to use an antiseptic solution to clean the skin at the time a CVC is inserted, at the moment we do not know which is the best antiseptic to use for babies. Currently in the UK many different antiseptics are being used in neonatal units and this is because we simply don't yet know which one works best or is safest to use for premature babies.

Our study is therefore comparing two commonly-used antiseptics for CVC insertion in premature babies. This is a small initial study (called a 'feasibility study') that will help us get some vital information about the use of these antiseptics in premature babies that will help us properly design a larger future study.

Does my baby have to take part?
It is entirely up to you whether you wish your baby to take part in this study. If you don't want your baby to take part, you do not have to give any reasons and neither your care nor your baby's will be affected. If you do take part, then you are still free to withdraw your baby from the study at any time, without giving any reason and without on-going care being affected.

What will happen if my baby joins the study?
If you consent to your baby taking part in this study, your baby will be randomly assigned to have their skin cleaned with either an alcohol-based antiseptic (70% isopropyl alcohol and 2% chlorhexidine) or a water-based antiseptic (2% chlorhexidine aqueous). The antiseptic used in your baby will be selected randomly by a special computer programme and will be used to disinfect your baby's skin both when their CVC is inserted and when it is removed. Neither you nor the staff caring for your baby will know which antiseptic your baby has been allocated.

After the CVC has been inserted, your baby will remain in the study until 2 days after its removal. During the study we will check daily for any skin reactions to the antiseptic. Also as part of this study, when the CVC gets removed we will take two skin swabs from the CVC insertion site to check for any bacteria and we will also send two small portions of the CVC itself away to the microbiology laboratory to check how many bacteria may have collected onto it. This will give us some initial information about how effective these two antiseptics may be at preventing CVC colonisation by skin bacteria.

Apart from the antiseptic used to clean your baby's skin and the skin swabs and catheter segments taken at CVC removal, all other aspects of the CVC insertion and removal will be completely in line with standard clinical management. No extra blood tests are required for this study. Being in the study will not interfere in any other way with the medical or nursing care your baby receives.

Catheters are usually removed because they are no longer needed (i.e. when a baby has reached full milk feeds and no longer needs direct liquid feeding into their bloodstream). But in about a quarter of babies who have a CVC in place, some signs of possible infection may develop before the CVC is due to be removed. If this happened to your baby while taking part in this study we would follow standard clinical practices, namely we would usually aim to remove the CVC early, send some routine blood tests to check for infection, and start your baby on antibiotics. But in addition, we will still send the study skin swabs and catheter

segments at CVC removal, because these may provide important additional information that will help us to know definitively whether your baby had developed a catheter-related infection. They will also allow us to check that any antibiotics your baby got started on were the right ones needed.

This study has been designed so that there is more chance of a baby receiving the alcohol-based antiseptic (3:1 ratio). The alcohol-based version is the commonest antiseptic being used in babies in the UK at present. Although we are interested to see how many catheters get colonised with bacteria with use of each of the two antiseptics being studied, the results from the alcohol-based antiseptic group will in particular help us know how many babies are needed for the large future study. This is why for this initial study any baby who participates is, on average, more likely to be allocated to the alcohol-based antiseptic. At the moment we don’t know whether alcohol is an important component of the antiseptic in babies. The large future study will answer this question.

What happens to the study samples?
The skin swabs and catheter segments will be sent to the local microbiology laboratory of this hospital for initial testing. Any baby who has a blood sample taken for suspected infection during the study would also have this sample tested in this local microbiology laboratory, as per usual clinical practice. If any of these samples turn out to be positive on testing, i.e. show growth of bacteria, then a specimen of the bacteria grown (called an ‘isolate’) will be stored for more detailed testing at a later date. The further testing will be done after sending the isolates away to a specialist microbiology laboratory at the University of East Anglia where the exact species of bacteria will be identified with the help of advanced tests and equipment. All isolates sent away to the University laboratory will be fully anonymised, and identifiable only through linking to our database by the study number. Positive isolates will be kept for a period of 2 years after completion of the study before being destroyed.

Are there any possible risks or side effects of taking part?
Both antiseptic solutions chosen for use in this study are already commonly used in premature babies in Europe and America. Skin reactions such as redness and chemical burns have occasionally been reported with both of these antiseptics. The risk of skin reactions is increased when excess antiseptic solution is used or when it is in prolonged contact with the skin in very premature babies. Our study aims to minimise the risk of any reactions by carefully limiting the use of antiseptic to the minimum needed to cover the skin and by avoiding any prolonged contact of antiseptic with the baby’s skin. We will check the skin closely for any reactions.

Skin reactions resulting from these two antiseptics appear to be uncommon in premature babies: one recent study from Ireland reported skin reactions in only 3 (2%) out of 148 premature babies treated with alcohol-based 2% chlorhexidine before CVC insertion; another study from Canada showed no adverse skin effects in any of the 199 premature babies whose skin was cleaned with the very same two skin antiseptics that we are now studying.

Are there any potential benefits to my baby from taking part?
We do not know if your baby will benefit directly from taking part in this study. If the antiseptic they receive in this study is more effective than the one normally used for CVC insertion, then they may benefit from a decreased risk of catheter infection. However this study is not designed to prove such possible benefit. The information we get from this initial study will nevertheless be important because it will help us to design a bigger study in the future. The future big study will test these same two antiseptics in a large number of babies to find out which one may be better. Doctors and nurses will then have a much better idea which antiseptic they should choose for skin preparation before CVC insertion in premature

babies to reduce the risk of catheter infection. We believe that the information from this initial study may therefore help towards improving the future care of premature babies.

Will taking part in this study be kept confidential?

Yes. At the beginning of the study your baby will be given a study number. This number (and not their name) will be used when studying and analysing the data. Information collected on your baby for this study will be entered electronically into a linked anonymised, password-protected, computer database, accessible by the researchers only. Your baby's involvement in this study will be noted in his/her medical records. With your permission we will let your GP know that your baby has taken part in this study. This will be done by including brief details about the study on the clinical summary that gets sent to your GP when your baby is discharged home from the neonatal unit.

With your consent we will collect some personal data to enable us to contact you in the future with the results of the research. Personal details will be kept for a period of no less than 10 years and will be kept only by the study organisers based in Norwich. At all times personal data will be held securely and will not be used for any other purpose. All other information collected for this study may also be used to help in future research studies but will never identify you or your baby. At all times the details will be handled only by authorised individuals and will remain confidential. Clinicians and research staff directly involved in the study will have direct access to your baby's medical records. In addition, employees of the Sponsor and/or the hospital Research and Development department and representatives from the Medicines and Healthcare products Regulatory Agency (MHRA) may require access to your baby's records as part of the monitoring procedures that are in place to oversee the conduct of the study. The full research records for this study will be retained for a period of not less than 25 years.

What will happen to the results of the research?

The results of this study will be shared with other doctors and nurses around the world who look after premature babies, so that they will be able to learn from the new information that might help them to improve their practice and care for babies. To do this a report containing the results of this study will be written, presented at scientific meetings and published in medical journals. Your baby's identity will not be made known in any circumstances.

Who has reviewed this study?

Before any research can go ahead in the NHS it needs to be checked by an independent group of people called a Research Ethics Committee. Their job is to ensure that any proposed research is ethical and to protect the safety, rights, well-being and dignity of participants. This study has been reviewed and approved by East of England – Cambridge South Research Ethics Committee, and also by the Research and Development department of this hospital. The study was also reviewed by the Neonatal Clinical Studies Group of the UK Medicines for Children Research Network (MCRN), and by Bliss (the premature baby charity). Some parents of premature babies that we previously cared for who required a CVC also helped us to design the study.

Insurance and remuneration

Your baby is covered by NHS insurance for any problems that may arise due to the study. If you consent for your baby to take part in this study you will not receive any payment or remuneration for their participation.

What if new information becomes available during the study?

We will discuss with you any important new information that may become available during the study which could affect your decision to let your baby take part.

Who is organising and funding the study?
The study is sponsored and by Norfolk and Norwich University Hospitals NHS Foundation Trust. The study has been funded by the National Institute for Health Research (NIHR) Research for Patient Benefit Programme.

What if I have any further questions or concerns?
If you have any questions or would like further information please ask one of the doctors or nurses caring for your baby, or alternatively please contact the doctor leading this study at this hospital directly:

Local Principal Investigator: *(add contact details)*

If you have any concerns about this study or the way it has been carried out, you should first contact your local study doctor named above who will do their best to address your queries. If you remain unhappy, you may contact your local Patient Advice and Liaison Service (PALS) for advice:

PALS: *(add contact details)*

What do I do now?
If you agree to your baby participating in this study, and have had all your questions answered satisfactorily, please complete and sign the consent form on the next page. You will be given a copy of the signed form to keep, along with this information sheet.

Thank you for reading this leaflet
and for thinking about taking part in this study

Maternal Consent Form

Please complete in black ballpoint pen

Hospital name: ____ **Study Number:** ☐☐☐

Baby's first name	Baby's last name

Title of study: ARCTIC Trial
Formal Title: Antiseptic Randomised Controlled Trial for Insertion of Catheters
Chief Investigator: Dr Paul Clarke

Please initial box

1. I confirm that I have read and understood the information in the Parental Informed Consent Form for this study (v 1.2, 18th November 2016) and have had the opportunity to consider the information, ask questions, and have had these answered satisfactorily. ☐
2. I understand that participation in this study is voluntary and that I am free to withdraw my baby from the study at any time without giving any reason, and without my or my baby's present or future medical care or legal rights being affected. ☐
3. I understand that relevant sections of medical notes and data collected during the study relating to me and my baby may be looked at by the researchers and by the Sponsor, Funder, regulatory authorities or my NHS Trust. I give permission for these individuals to have access to these notes where it is relevant to taking part in this research. ☐
4. I agree that my personal contact details can be collected, stored, and sent to the co-ordinating centre in Norwich, along with a copy of this signed consent form, to enable follow-up contact with me regarding this study. This is on the understanding that any such information will be treated confidentially. ☐
5. I understand that information held and managed by the Health and Social Care Information Centre and other central UK NHS bodies may be used to help contact me in the future or to provide information about my baby's health status. I also agree that data collected in this study may be used to help in future research studies. ☐
6. I agree to my GP being informed of my baby's participation in the study. ☐
7. I agree that my baby may take part in the ARCTIC study. ☐

Mother's Name	Name of health professional taking consent
Mother's Signature	Signature
D D / M M / Y Y	D D / M M / Y Y

EudraCT No. 2015-000874-36. REC Ref. 15/EE/0345
Protocol Version 3.0, 18th November 2016

This project was funded by the National Institute for Health Research Research for Patient Benefit (RfPB) Programme (Project reference: PB-PG-1013-32076)

NPEU Clinical Trials Unit, Nuffield Department of Population Health
University of Oxford, Old Road Campus, Headington, Oxford, OX3 7LF
T: 01865 289700 F: 01865 289701
E: arctic@npeu.ox.ac.uk www.npeu.ox.ac.uk/arctic
