# Supplementary material for: Safety and efficacy of 2% chlorhexidine gluconate aqueous versus 2% chlorhexidine gluconate in 70% isopropyl alcohol for skin disinfection prior to percutaneous central venous catheter insertion in preterm neonates: the ARCTIC randomised-controlled feasibility trial protocol
Source: BMJ Open. 2019 Feb 8;9(2):e028022. doi: 10.1136/bmjopen-2018-028022 (PMC6411256; doi:10.1136/bmjopen-2018-028022)
Supplement: Author's manuscript [file bmjopen-2018-028022.draft_revisions.pdf]

BMJ Open

BMJ Open is committed to open peer review. As part of this commitment we make the peer review history of every article we publish publicly available.

When an article is published we post the peer reviewers' comments and the authors' responses online. We also post the versions of the paper that were used during peer review. These are the versions that the peer review comments apply to.

The versions of the paper that follow are the versions that were submitted during the peer review process. They are not the versions of record or the final published versions. They should not be cited or distributed as the published version of this manuscript.

BMJ Open is an open access journal and the full, final, typeset and author-corrected version of record of the manuscript is available on our site with no access controls, subscription charges or pay-per-view fees (http://bmjopen.bmj.com).

If you have any questions on BMJ Open's open peer review process please email editorial.bmjopen@bmj.com

BMJ Open

# Safety and efficacy of 2% chlorhexidine gluconate aqueous versus 2% chlorhexidine gluconate in 70% isopropyl alcohol for skin disinfection prior to percutaneous central venous catheter insertion in preterm neonates: the ARCTIC randomised-controlled feasibility trial protocol

| | |
|---|---|
| Journal: | *BMJ Open* |
| Manuscript ID | bmjopen-2018-028022 |
| Article Type: | Protocol |
| Date Submitted by the Author: | 19-Nov-2018 |
| Complete List of Authors: | Clarke, Paul; Norfolk and Norwich University Hospitals NHS Foundation Trust, Neonatal intensive Care Unit; University of East Anglia, Norwich Medical School<br>Craig, Jean; University of East Anglia, Research Design Service, Norwich Medical School<br>Wain, John; University of East Anglia Faculty of Medicine and Health Sciences, Norwich Medical School; Quadram Institute Biomedicine<br>Tremlett, Catherine; Norfolk and Norwich University Hospitals NHS Foundation Trust, Department of Microbiology<br>Linsell, Louise; University of Oxford, National Perinatal Epidemiology Unit, Nuffield Department of Population Health<br>Bowler, Ursula; University of Oxford, National Perinatal Epidemiology Unit, Nuffield Department of Population Health,<br>Juszczak, Ed; University of Oxford, National Perinatal Epidemiology Unit, Nuffield Department of Population Health,<br>Heath, Paul; St George's University of London, Paediatric Infectious Diseases Research Group, Infection and Immunity, |
| Keywords: | antiseptic, biocide, disinfection, central line associated bloodstream infection, catheter |
| | |

SCHOLARONE™
Manuscripts

**TITLE**

# Safety and efficacy of 2% chlorhexidine gluconate aqueous versus 2% chlorhexidine gluconate in 70% isopropyl alcohol for skin disinfection prior to percutaneous central venous catheter insertion in preterm neonates: the ARCTIC randomised-controlled feasibility trial protocol

**Authors**

Paul Clarke[1,2] (Corresponding Author)

Jean Craig[3]

John Wain[2,4]

Catherine Tremlett[5]

Louise Linsell[6]

Ursula Bowler[6]

Ed Juszczak[6]

Paul T. Heath[7]

1. Neonatal Unit, Norfolk and Norwich University Hospitals NHS Foundation Trust, Colney Lane, Norwich, Norfolk, NR4 7UY, UK.

2. Norwich Medical School, University of East Anglia, Norwich, UK

3. Research Design Service, Norwich Medical School, University of East Anglia, Norwich, UK

4. Quadram Institute Bioscience, Norwich Research Park, Norwich, UK.

5. Department of Microbiology, Norfolk and Norwich University Hospitals NHS Foundation Trust, Norwich, UK

For peer review only



For peer review only - http://bmjopen.bmj.com/site/about/guidelines.xhtml

6. National Perinatal Epidemiology Unit, Nuffield Department of Population Health, University of Oxford, Oxford OX3 7LF

7. Paediatric Infectious Diseases Research Group, Infection and Immunity, St. George's University of London, London, UK

[1.]Corresponding Author: Prof Paul Clarke paul.clarke@nnuh.nhs.uk Tel. +44 1603 286342

For peer review only

**Word count: 4238**



For peer review only - http://bmjopen.bmj.com/site/about/guidelines.xhtml

## ABSTRACT

**Introduction:** Catheter-related sepsis is one of the most dangerous complications of neonatal intensive care and is associated with significant morbidity and mortality**.** Use of catheter-care 'bundles' has reduced the incidence of catheter-related sepsis, though individual components have not been well studied. Better evidence is needed to guide selection of the most appropriate antiseptic solution for skin disinfection in preterm neonates. This study will inform the feasibility and design of the first randomised controlled trial to examine the safety and efficacy of alcohol-based versus aqueous-based chlorhexidine antiseptic formulations for skin disinfection prior to percutaneous central venous catheterisation in preterm neonates. The antiseptics to be compared are 2% CHG aqueous and 2% CHG in 70% isopropyl alcohol.

**Methods and Analysis:** The Antiseptic Randomised Controlled Trial for Insertion of Catheters (ARCTIC) is a two-centre randomised-controlled feasibility trial. At least 100 preterm infants born at <34 weeks' gestation and due to undergo percutaneous insertion of a central venous catheter will be randomly allocated to receive prior skin disinfection with one of the two antiseptic solutions. Outcomes include: i) recruitment and retention rates; ii) completeness of data collection; iii) numbers of enrolled infants meeting case definitions for definite catheter-related sepsis, catheter-associated sepsis, and catheter colonisation; and iv) safety outcomes of skin morbidity scores recorded daily from catheter insertion until 48 hours post removal. The key feasibility metrics will be reported as proportions with 95% confidence intervals. Estimated prevalence of catheter colonisation will allow calculation of sample size for the large-scale trial. The data will inform whether it will be feasible to progress to a large-scale trial.




For peer review only - http://bmjopen.bmj.com/site/about/guidelines.xhtml

**Ethics and dissemination:** ARCTIC has been approved by the National Health Service Health Research Authority National Research Ethics Service Committee East of England (Cambridge South) (IRAS ID 163868), was adopted onto the National Institute of Health Research Clinical Research Network portfolio (CPMS ID 19899), and is registered with an International Standard Randomised Control Trials Number (ISRCTN: 82571474) and European Clinical Trials Database (EudraCT) number 2015-000874-36. Dissemination plans include presentations at scientific conferences, scientific publications, and sharing of the findings with parents via the support of Bliss baby charity.

**Registration details:** Trial registration numbers ISRCTN82571474; EudraCT No. 2015-000874-36.

**Keywords**

Antiseptic; disinfection; sepsis; central line associated bloodstream infection; trial

**Article Summary**

**Strengths and limitations of this study**

- The ARCTIC study will be one of only very few randomised controlled trials of skin antiseptics in preterm neonates and the first to compare aqueous 2% chlorhexidine gluconate versus 70% isopropyl alcohol-based 2% chlorhexidine gluconate for cutaneous disinfection prior to central venous catheterisation
- The trial will collect rigorous, prospective safety data following antiseptic application through daily skin safety assessments using a validated neonatal skin scoring tool



For peer review only - http://bmjopen.bmj.com/site/about/guidelines.xhtml

- This will be the first study in neonates to undertake molecular typing of isolates to verify that skin-colonising and blood-cultured organisms match catheter-colonising organisms to a species level in babies with suspected sepsis, thus allowing definitive proof of catheter-related sepsis
- The target sample size is based upon an anticipated incidence of catheter colonisation of 20% in the reference antiseptic group, estimated with a 95% Confidence Interval of 11% to 31%, and is not powered to detect differences in clinical outcomes
- This trial will show whether a future large-scale multicentre randomised controlled non-inferiority trial of the same antiseptics is feasible and will determine the sample size required for such a trial

For peer review only



For peer review only - http://bmjopen.bmj.com/site/about/guidelines.xhtml

**Introduction**

Percutaneously-inserted central venous catheters (PCVCs) are inserted daily in neonatal intensive care units (NICUs) across the world to deliver hyperosmolar parenteral nutrition solutions to preterm neonates. PCVCs may remain in situ for weeks[1], but their presence entails a major risk for bloodstream infection. In a previous study, 32% of inserted PCVCs were colonised with potentially-pathogenic bacteria at the point of removal, and 8% overall were associated with definite catheter-related sepsis (CRS).[1] Extraluminal colonisation is the main route of catheter colonisation in short-term CVCs: skin bacteria traverse the catheter insertion site onto the catheter, colonise the line, and act as focus for CRS.[2] In one study the presence of skin bacteria at the catheter exit site was associated with an 8-fold increased risk of catheter colonisation and a 10-fold increased risk of CRS caused by the same organism.[2]

For preterm babies in the NICU, CRS is a dangerous complication associated with significant morbidity and mortality**.** Sepsis increases the duration of intensive care and hospitalisation, need for antibiotics, and risks for adverse neurodevelopmental outcome**.** Coagulase-negative Staphylococcal infections cause the majority of CRS in the NICU (80-90%), may be life-threatening, and can cause permanent lifelong injury and disability in survivors, including cerebral palsy.[3,4]

Reduction of CRS has been a major goal of the National Health Service for the past decade.[5] Catheter-care ‘bundles’, guidelines incorporating collected good practices for catheter insertion and maintenance, have successfully reduced the incidence of catheter colonisation and CRS in the NICU,[6] though await universal adoption.[7]



For peer review only - http://bmjopen.bmj.com/site/about/guidelines.xhtml

The individual components of catheter-care bundles have not been well studied in randomised-controlled trials (RCTs). Adequate skin disinfection of the catheter insertion site is arguably the most important component of catheter-care bundles to prevent catheter colonisation and CRS. Optimal skin preparation will abolish or significantly reduce numbers of skin organisms, so limiting risks of residual skin colonisation by bacteria that may then colonise a PCVC and cause CRS.

Studies in adults, including meta-analysis, show that alcohol-based antiseptics are superior for topical antisepsis,[8,9] and UK national evidence-based guidelines recommend use of 2% chlorhexidine (CHG) in 70% isopropyl alcohol for skin antisepsis in adults and older children. However there is no guidance for preferred antiseptic in infants, including preterm infants, due to the lack of evidence and specific safety concerns in this population.[10,11] The best antiseptic to use for preterm babies is still unknown, and multiple different antiseptics, combinations, and concentrations are presently being used in UK NICUs; approximately half using a 2% concentration of CHG and 60% an isopropyl alcohol-containing CHG formulation.[12] For preterm neonates, there is no Cochrane review comparing skin antiseptics for cutaneous disinfection prior to PCVC insertion, and only two RCTs have compared topical antiseptics for PCVC insertion.[13,14]

There are risks associated with antiseptic use peculiar to preterm infants. Their thin skin is vulnerable to chemical injury and absorption. Chemical skin burns have been described with all the currently-used topical antiseptics, including both aqueous and alcohol-containing CHG formulations, and iodine solutions,[15] as well as with octenidine.[16] Topical alcohol use may also increase the risk of systemic chemical absorption.[17]



For peer review only - http://bmjopen.bmj.com/site/about/guidelines.xhtml

There are no published RCTs that have examined the safety and efficacy of alcohol-based versus aqueous CHG formulations for neonatal antisepsis. This feasibility study aims to inform the safety and assist the design and planning of a future large-scale multicentre RCT that will examine whether 2% CHG aqueous is non-inferior in antiseptic efficacy compared to 2%CHG-70%IPA for skin disinfection prior to PCVC insertion in preterm neonates. An aqueous CHG is likely to have fewer side effects than an alcohol-based CHG, and would therefore be preferable if found to be non-inferior in terms of antisepsis.

## OBJECTIVES

To determine the proportion of babies in the 2%CHG-70%IPA group with colonisation of at least one of the two catheter segments taken at catheter removal. Catheter colonisation will be the primary outcome in the full-scale trial because it is a valid surrogate for CRS;[2] to determine factors affecting recruitment and process outcomes that will help refine the design of the large-scale trial; also to estimate numbers of enrolled infants who have definite CRS and numbers with catheter-associated sepsis, determine suitability and completeness of data collection methods, and describe any skin morbidity occurring in trial participants related to use of the study antiseptics.



For peer review only - http://bmjopen.bmj.com/site/about/guidelines.xhtml

## Methods and analysis:

### Study design

A feasibility, masked randomised controlled trial of investigational medical products (IMPs). Preterm infants born at <34 weeks' gestation who are due to undergo planned insertion of a PCVC will be randomised to receive one of two topical disinfection agents for skin antisepsis: 2% chlorhexidine gluconate aqueous (2%CHG-aqueous), or 2% chlorhexidine gluconate in 70% isopropyl alcohol (2%CHG-70%IPA).

### Study Setting

Two tertiary-level neonatal units in the UK, Norfolk and Norwich University Hospital and Medway Maritime Hospital, which each cater to a total of 5000-6000 deliveries per year.

### Eligibility

**Inclusion criteria**

- Preterm infants born at <34 weeks' gestation
- Requiring routine insertion of a PCVC for parenteral nutrition
- No new episode of suspected sepsis with commencement of antibiotics occurring within the 48 hours preceding planned catheter insertion
- No other indwelling PCVC already in situ

**Exclusion criteria**

- No realistic prospect of survival in the short term
- Life-threatening congenital abnormality
- Underlying skin condition



For peer review only - http://bmjopen.bmj.com/site/about/guidelines.xhtml

- Another indwelling PCVC already in situ or previously enrolled into the study
- Positive blood culture (BC) within the past 7 days without a subsequent negative BC result
- Antibiotic treatment commenced for suspected sepsis within the preceding 48 hours

**Key Definitions**

*Definite catheter-related sepsis* (CRS): a peripheral BC plus any catheter segment (i.e. one of the ~1cm long proximal or tip catheter portions) positive with the same organism, based on bacterial culture, antibiotic sensitivity and molecular typing, from a neonate who had an indwelling PCVC and clinical signs of sepsis but no other focus of sepsis

*Catheter colonisation*: a catheter that at the time of removal has either one or both segments that are culture positive.

*Catheter-associated sepsis*: a baby with clinical signs of sepsis and an accompanying positive BC in the period between catheter insertion and 48 hours post removal but who has no other focus of sepsis and in whom both catheter segment cultures are negative

**Recruitment**

Preterm babies potentially suitable for the trial will be identified by the clinical healthcare teams. Parents of such infants will be approached for consent by the research team or delegated suitably-qualified member of the clinical healthcare team trained in study procedures and Good Clinical Practice (GCP). A written parent information sheet that forms part of the parental Informed Consent Form will be provided to help explain the study. Written maternal consent will be obtained and countersigned by the person who obtained informed consent (principal investigator [PI], or appropriately qualified healthcare professional with delegated authority).



For peer review only - http://bmjopen.bmj.com/site/about/guidelines.xhtml

**Randomisation**

Following consent, randomisation to either 2%CHG-70%IPA antiseptic or 2%CHG-aqeuous antiseptic will take place as close as possible to the time of planned catheter insertion. Randomisation will be managed via a secure web-based facility hosted by the National Perinatal Epidemiology Unit Clinical Trials Unit (CTU) and will use a 3:1 allocation ratio in favour of the 2%CHG-70%IPA antiseptic group, the group that will inform the power calculation for the large-scale trial. Groups will be stratified by birth gestation (<28 weeks; 28 weeks+0 days to 33 weeks+6 days) and by centre. Treatment allocation will be masked such that the allocation will not be known by clinicians, the baby's family, laboratory staff, or trial outcome assessors.

**Interventions**

The Trial procedures are summarised in **Figure 1.**

**Skin Disinfection at Catheter Insertion**

Study packs stored in a locked, secure, temperature-monitored cupboard will contain two bottles of the same allocated antiseptic IMP each labelled with the same identifying code. One bottle will be opened at catheter insertion, the other will be retained for use at catheter removal. The disinfection procedure requires sparing application of allocated antiseptic solution for 10-20 seconds from sterile gauze to the skin site area selected for catheterisation. Instructions are: use only the minimal volume of antiseptic necessary for skin coverage, avoid any pooling of antiseptic, ensure that any excess solution and any



For peer review only - http://bmjopen.bmj.com/site/about/guidelines.xhtml

soaked drapes or gowns are removed to avoid any prolonged contact with the skin, and allow the disinfected area to air dry completely (for ≥30 seconds) before proceeding with catheter insertion. Excepting in the case of failed catheterisation, saline or water must not be used to wipe the disinfected skin area following application of antiseptic solution.

**Catheter Insertion**

Catheter insertion will be standardised, using a working guideline common to both participating centres that requires strict aseptic technique and encompasses established good clinical practices for PCVC insertion and care adopted from catheter-care bundles.[5, ,18,19] The decision to insert a PCVC and choice of catheter (Premicath 1Fr/28G or Epicutaneo-Cava catheter 2Fr/24G: both Vygon UK Ltd, Cirencester, UK) is at the discretion of the attending clinical team. All personnel involved will be trained in catheter insertion and maintenance procedures. An insertion checklist will be completed for all catheterisations and a disposable face mask will be worn by the operator inserting the catheter for asepsis purposes and also to minimise the risk of possible unblinding from any smell of alcohol.

**Assessment of Skin Condition**

Skin status will be recorded using a validated neonatal contact dermatitis scoring system, the Neonatal Skin Condition Score,[20] with minor modification. Assessments will be undertaken by a nurse trained in use of the scoring system and will be recorded at baseline (prior to application of antiseptic), within 10-30 minutes after catheter insertion, and then daily until 48 hours following catheter removal, or daily for 48 hours after antiseptic application in cases where catheterisation is unsuccessful. Serious chemical skin burns



For peer review only - http://bmjopen.bmj.com/site/about/guidelines.xhtml

adjudged by a PI or delegate to be severe or moderately severe, will be notified to the Medicines and Healthcare Products Regulatory Agency (MHRA).[21]

**Catheter Removal and obtainment of Study specimens**

Catheters are usually removed when no longer required though sometimes removal is warranted earlier than intended because of complications, including suspected sepsis. If a catheter is being removed from a baby with suspected sepsis then a concurrent peripheral BC will be obtained as per routine clinical practice. The decision for catheter removal for enrolled babies lies with the attendant clinical team.

The following samples will be obtained at the time of catheter removal for microbiological analysis:

i) Two exit site skin swabs taken *before* catheter removal: the first after removing the transparent covering dressings but prior to skin disinfection; the second taken post new disinfection of the insertion site, once the antiseptic has dried. This disinfection procedure is intended to limit the risk of catheter contamination by residual skin organisms during the removal of the catheter and will use the same allocated antiseptic as was used for catheter insertion. Both specimens will be obtained by rolling the swab tip several times across the skin of the catheter insertion site, over an area within <0.5 cm radius of the insertion site but also including the actual puncture site.

ii) Two approximately 1 cm long catheter segments (proximal and tip) *after* catheter removal. Proximal catheter segments have higher colonisation rates than tips,[1] therefore microbiological analysis of both catheter segments rather than the tip alone may improve the diagnostic yield of catheter colonisation.



For peer review only - http://bmjopen.bmj.com/site/about/guidelines.xhtml

Catheter removal requires two trained persons and care to avoid cross-contamination between segments while sectioning the catheter. Two separate sets of sterile forceps, two pairs of sterile scissors, and two sterile pre-labelled universal containers are required. Before removing the catheter, the subcutaneous insertion length will be noted from the external catheter markings. The catheter will be removed onto a sterile paper towel field then sectioned using separate pairs of sterile scissors to obtain two ~1 cm-long formerly subcutaneous catheter segments: i) tip, and ii) a proximal segment, taken approximately 1-2 cm distal to the point of skin entry (**figure 2**). The individual segments will be placed into separate appropriately-labelled sterile universal containers using separate pairs of sterile forceps.

**Microbiology**

The catheter segments and skin swabs will be submitted to the local microbiology laboratory for routine culture and antibiotic sensitivities. BCs sent from babies with suspected sepsis at the time of catheter removal will undergo standard culture methods. Bacterial growths from skin swab cultures will be assessed using a semi-quantitative method.[22] All laboratory staff will be blinded to antiseptic allocation. Isolates from culture-positive skin swabs, blood, and catheter segmental cultures will be retained for molecular typing. Initial identification of organisms will be done by Mass Spectrometry. Those giving similar patterns will be analysed using Next Generation sequencing using a multiplexed approach on the Illumina MiSeq. Molecular typing of paired blood and catheter isolates from the same baby will allow confirmation that isolates are identical to a species level, for definitive diagnosis of definite CRS. While few post-disinfection skin swabs are



For peer review only - http://bmjopen.bmj.com/site/about/guidelines.xhtml

expected to be positive, molecular typing of skin swab isolates will be done for any babies with colonised catheters: isolation of paired identical species could indicate possible catheter contamination by skin organisms during catheter removal. Babies with positive BCs will be managed according to local clinical guidelines; involvement in this trial will not dictate or influence clinical antibiotic prescriptions.

**Outcome measures**

I. Proportion of babies in the 2%CHG-70%IPA arm with catheter colonisation as determined by positive bacterial culture from one or both of the catheter segments taken at catheter removal (primary outcome)

II. Rates of recruitment and retention to the study, and the collection of views of parents and clinicians on factors affecting recruitment and retention

III. Proportion of infants with positive exit-site skin swabs at catheter removal

IV. Number and type of catheter segments culture positive at removal

V. Bacterial species (typed via molecular methods) of isolates identified on positive BC, exit-site skin swabs, and catheter segments

VI. Proportion of infants undergoing an infection screen in the period between catheter insertion and 48 hours post catheter removal that meets case definition for definite CRS

VII. Proportion of infants with positive blood culture from any infection screen in the period between catheter insertion and 48 hours post-catheter removal that meets definition for catheter-associated sepsis

VIII. Rate of CRS per 1000 PCVC days

IX. Rate of catheter-associated sepsis per 1000 PCVC days



For peer review only

X. Proportion of infants completing study with complete data for primary outcome and proportions of infants with missing data collection forms

XI. Daily skin morbidity scores in the period between catheter insertion and 48 hours post-catheter removal, and in the period between antiseptic application and 48 hours post antiseptic application where catheterisation was unsuccessful.

**Supportive care of participants**

The clinical management of babies enrolled in the study will follow standard local practices. For the purposes of this feasibility study, if CRS is suspected the ideal will be catheter removal at that time. However, it is recognised that a pragmatic approach is sometimes needed, especially for very premature babies in whom catheter replacement may be difficult and challenging. Efforts will be made to minimise local differences in treatment practices between sites through training.

**Discontinuation of Trial intervention**

The trial intervention will be stopped on parental request, or if the baby develops serious adverse skin damage that, in the opinion of the responsible PI, was caused by the IMP. Thus, if any baby has a clinically-significant chemical skin burn following IMP application at catheter insertion then the allocated antiseptic will be withheld from use for skin disinfection at catheter removal. In such instance skin swab and catheter sections will still be obtained as per removal procedure and the protocol deviation will be recorded.

**Blinding and unblinding**

The antiseptic IMPs will be manufactured by a MHRA-accredited Specialist Pharmacy



For peer review only - http://bmjopen.bmj.com/site/about/guidelines.xhtml

Manufacturing Unit compliant with Good Manufacturing Practices. The IMP will be supplied in bottles and both products will be coloured pink (using carmoisine) and visually indistinguishable. To maintain blinding, each baby will be issued a unique allocation number corresponding to the study pack number. Emergency unblinding for valid medical or safety reasons is via the randomisation website using a single-use access code provided in a sealed envelope in the Investigator Site File.

**Sample size**

Our previous study found that 32% of PCVCs had a colonised tip and/or proximal segment at removal after using much weaker concentration (0.015%-0.05%) CHG solutions and the definite CRS rate was 6.8 per 1000 catheter days.[1] In comparison, a UK NICU that routinely used 2%CHG-70%IPA for PCVC insertions reported a 34% lower CRS rate (4.5 per 1000 catheter days).[18] Presuming that alcohol-based 2%CHG is the major factor of benefit in catheter care, by extrapolation we might reasonably expect to see a 34% reduction in extraluminal catheter colonisation rates by using 2%CHG-70%IPA solution. Thus we estimate a catheter colonisation rate of approximately 20% (0.66 x 32% = 21%) may be achieved with 2%CHG-70%IPA solution. A 3:1 allocation ratio in favour of the reference 2%CHG-70%IPA group requires a target sample size of approximately 93 babies with successfully inserted (and removed) catheters (i.e. n=70 in the reference group) to estimate the critical parameters for a future, large-scale trial with an adequate degree of precision. With this target sample size, the anticipated incidence of the primary outcome in the reference group of 20% will be estimated with a 95% Confidence Interval of 11% to 31%. With a sample size of 93 babies with successfully inserted catheters, the anticipated recruitment/uptake rate of 75%[13] will be estimated with a 95% Confidence Interval of 0.65 to 0.83. A sample size of



For peer review only - http://bmjopen.bmj.com/site/about/guidelines.xhtml

approximately 93 babies having catheters successfully inserted/removed will require parents of at least 124 eligible babies to be approached.

**Data management and analysis**

Outcome data include routinely recorded clinical information obtainable from clinical and local microbiological laboratory records. Data verifying species of catheter colonisation will be collected following further analysis of positive isolates by molecular typing. Data will be collected using study-specific data collection forms for: Trial Entry and Randomisation, Main Outcome Data (catheter insertion, skin condition assessment, sepsis evaluation, and antimicrobial therapy), Catheter Removal, Microbiology Data, Unsuccessful Catheterisation Episodes, Discontinuation of Intervention, Withdrawal, and Foreseeable Serious Adverse Events (SAE). In addition, information will be collected and reported to the sponsor using the sponsor's SAE report Form and Incident Form, to report any deviation from the protocol, trial-specific procedures, or GCP.

Data collection will proceed from randomisation until 48 hours post catheterisation for successfully inserted catheters, or until 48 hours after last antiseptic application for unsuccessful catheterisation. If a baby is discharged from its recruiting centre before study completion, to try to achieve complete follow-up safety data, the research team will contact the receiving clinical nursing team to request routine daily documentation regarding status of catheter insertion site skin and details of any new clinical sepsis events until 48 hours post catheter removal.

All data will be collected, transferred, and stored in compliance with GCP and current Data



For peer review only - http://bmjopen.bmj.com/site/about/guidelines.xhtml

Protection legislation. The trial co-ordinating centre (Norwich) will hold the main administrative database for the trial. Data acquired by the enrolling units will initially be recorded onto paper data collection forms, followed by entry into an OpenClinica database (OpenClinica, Waltham, USA) administered by the National Perinatal Epidemiology Unit (NPEU). Access to this database will be via a web browser and restricted to authorised users. The database has been tested and validated prior to use. All data collection, transfer, and storage will comply with GCP and Data Protection legislation.

**Statistical Analysis Plan**

A statistical analysis plan for proposed analysis and presentation of the results of the trial will be drawn up by the delegated CTU medical statistician. Drafts will be reviewed by CTU personnel, by the CI, and by the chair of the TSC and a final version will be approved prior to the end of recruitment. Any deviations from the plan will be described and justified in the final report. Analysis will be carried out by an appropriately qualified and experienced statistician, who will ensure the integrity of the data during their processing.

**Site Training**

Each recruiting centre is staffed by a local research nurse dedicated to support the study. Initiation visits at each participating neonatal unit will be performed by the CI and study Research Nurse, also attended by the sponsor's representative. Training in study-specific procedures and in awareness of the principles of GCP will be provided for nursing and medical staff in each site by the local PI and research nurses, who will also help maintain training and delegation logs.

For peer review only



For peer review only - http://bmjopen.bmj.com/site/about/guidelines.xhtml

**Monitoring**

The sponsor's nominated representatives will undertake monitoring visits during the course of the study at each recruiting site to check for completeness and quality of data collection and adherence to the study protocol and reporting requirements. A monitoring plan is in place to determine the frequency and scope of site monitoring based on continuing risk review. Face-to-face monitoring visits will initially be undertaken within the first 6 months and the frequency and mode of ongoing site monitoring will be revised following assessment of recruitment rates, number of data queries, and safety/incident reports.

**Pharmacovigilance**

Safety of participants will be assessed continuously from randomisation until 48 hours post catheter removal. The frequency of adverse events and SAEs as defined by The International Conference on Harmonisation and that would normally require reporting within a clinical trial is expected to be high in this population. In accordance with regulatory guidance which allows for exceptions in such circumstances, a modified reporting plan was approved by the research ethics committee and by the MHRA. This plan exempted the need for routine reporting of pre-specified SAEs that are a foreseeable occurrence in preterm babies, unless considered causally related to IMP or trial procedures. Unforeseeable SAEs will be reported on the sponsor's SAE form. The relationship of each adverse event to the trial medication will be determined by a medically qualified individual. All reportable SAEs with causality assessed as 'possibly', 'probably', or 'definitely', will be considered as related to IMP. All SAEs assigned by the PI or delegate (or following sponsor/CI review) as suspected to be related to IMP *and* unexpected will be classified as suspected unexpected serious adverse reactions and subject to expedited reporting to the MHRA.



For peer review only - http://bmjopen.bmj.com/site/about/guidelines.xhtml

**Data and safety monitoring**

The Data Monitoring Committee (DMC) is responsible for safeguarding the interests of the trial participants and making recommendations to the Trial Steering Committee (TSC). The ARCTIC DMC roles, responsibilities, and operating procedures are defined in the ARCTIC DMC Charter. It is composed of three independent multidisciplinary experts who are not involved in the conduct of the trial in any way. They met prior to the initiation of enrolment and determined a plan to review the protocol, compliance, safety, and outcome data after 50 babies had been recruited. The TSC is composed of 8 independent members and has a Charter defining members' roles and responsibilities. Its Chair and the majority of the TSC membership are independent of the trial. The TSC provides the overall supervision, monitors progress and conduct of the trial, and advises on its scientific credibility. The TSC will consider and act, as appropriate, upon any recommendations of the DMC and carries ultimate responsibility for deciding whether the trial needs to be stopped on grounds of safety or efficacy. The TSC will report on trial progress to the trial funder.

**Ethics and dissemination**

The trial received approval from the National Health Service Health Research Authority National Research Ethics Service Committee East of England (Cambridge South) (IRAS ID 163868). Clinical trial authorisation was granted by the MHRA (REF: 13630/0009/001-0001). Written approvals were received from individual hospital sites prior to recruitment. The Trial commenced recruitment under protocol version 3.0, dated 18th November 2016; full protocol is available at: https://www.npeu.ox.ac.uk/arctic. The investigator or a suitably qualified person designated by the local PI will obtain written informed consent from the



For peer review only - http://bmjopen.bmj.com/site/about/guidelines.xhtml

patient's parent/legally-accepted representative before any trial-specific activity is performed. The CI will ensure that the trial is conducted in accordance with the principles of the Declaration of Helsinki and in conformity with GCP. The trial's findings will be presented at national and international scientific meetings and conferences and will be published in an open access peer-reviewed journal.

**Patient and public involvement**

The study proposal benefitted from extensive patient and public involvement during its development. Advice received included regarding content of the Parental Informed Consent Form and aspects of protocol design. Input was received from Bliss baby charity (www.bliss.org.uk), by PPIRes (http://nspccro.nihr.ac.uk/public-and-patient-involvement-in-research), and by a consumer member representative of the Neonatal Clinical Specialty Group of the Medicines for Children Research Network. We also consulted with a local parent support group and with parents of babies who had suffered CRS. Two lay members are involved in trial management as members of the TSC, and will be involved in dissemination of findings.

**Conclusions**

Recruitment to ARCTIC commenced in March 2017 and the projected overall trial end date is 31/03/2019. It is hoped that the findings of this feasibility study will pave the way for the definitive large-scale efficacy/safety study. The anticipated large-scale study will be a multi-centre non-inferiority RCT of the same two antiseptics for skin disinfection prior to PCVC insertion in preterm neonates. Primary outcome will be catheter colonisation as determined by culture of one or more catheter segments taken at catheter removal. National evidence-



For peer review only - http://bmjopen.bmj.com/site/about/guidelines.xhtml

For peer review only

based guidelines for preventing healthcare-associated infections in the NHS, (‘epic’), commissioned by the Department of Health, were published in 2001, 2007, and 2014 to incorporate new research evidence.[10,11] Due to the lack of previous quality RCTs in the preterm population, no previous guidelines included advice or recommendations on antiseptics specific for preterm neonates. We anticipate that the findings from this research will be incorporated into a future version of the epic guidelines.

For peer review only



For peer review only - http://bmjopen.bmj.com/site/about/guidelines.xhtml

**Figure Legends:**

**Figure 1:** Trial procedures

**Figure 2:** Picture showing catheter sections taken at catheter removal

**Author Contributions:**

PC and PTH conceived the idea for this study. PC designed the study, wrote the protocol, and is Chief Investigator responsible for all aspects of the study including preparation and submission of the grant application, application for Clinical Trial Authorisation from the Licensing Authority (MHRA), securing funding, project management, data collection and preparation of the manuscript for publication. JC helped design the study and contributed to manuscript revision. PTH, JW, CT, LL, UB, and EJ helped design and refine the study, developed the protocol, and contributed to manuscript revision. LL and EJ in addition provided statistical and methodological expertise, and UB helped with the logistical aspects of the trial involving the trials unit. All authors approved the final version of the manuscript.

**Acknowledgments:** We thank Amy Nichols (research nurse) and Tracy Oliver (Study coordinator) at the Norwich site; Drs Aung Soe (PI), Dr Santosh Pattnayak (Co-Investigator) and Helen Harizaj (research nurse) at Medway site. The CI is grateful to Nigel Lambert and Susan Stirling for helpful early input into study design, and Andy King and Christopher Partlett of NPEU CTU for help with database, programming, and statistical support. We also sincerely thank Professor Kate Costeloe, TSC chair, and Dr Mark Turner, DMC chair, and the membership of these committees. PC wishes to acknowledge the support and help of Lisa Chalkley, Julie Dawson, Basia Brown, and Francesca Dockerty for the sponsor.



For peer review only - http://bmjopen.bmj.com/site/about/guidelines.xhtml

**Funding** The ARCTIC Trial is funded by the National Institute for Health Research Research for Patient Benefit Programme (Project ref: PB-PG-1013-32076).

**Sponsor** The Norfolk and Norwich University Hospitals NHS Foundation Trust is the sponsor of this trial. Sponsor contact details: R+D Manager, Research and Development Office, Level 3 East Block, Norfolk and Norwich University Hospitals NHS Foundation Trust, Colney Lane, Norwich, Norfolk, NR4 7UY. Tel. 01603 288437; Fax. 01603 289800; Email: rdoffice@nnuh.nhs.uk

**Competing interests** None declared.

**Disclaimer:** The Trial sponsor and funder (NIHR) had no role in trial design, and will have no role in gathering of data, access to data, interpretation of data, preparation of the manuscript, or decision to publish the results. The views expressed in this publication are those of the authors and not necessarily those of the NHS, NIHR or the Department of Health.

**Patient consent** Written maternal consent obtained for all participants.

**Ethics approval**: NHS Health Research Authority National Research Ethics Service Committee East of England (Cambridge South)

**Provenance and peer review:** Not commissioned; peer reviewed for ethical and funding approval prior to submission.

**Data sharing statement** Data collection is underway. The data supporting the findings of this study will be available in due course from the Chief Investigator upon reasonable request and with the permission of NPEU/sponsor.

**Open Access** This is an Open Access article distributed in accordance with the terms of the



For peer review only - http://bmjopen.bmj.com/site/about/guidelines.xhtml

Creative Commons Attribution (CC BY 4.0) license, which permits others to distribute, remix, adapt and build upon this work, for commercial use, provided the original work is properly cited. See: http://creativecommons.org/licenses/by/4.0/

**REFERENCES**

1. Ponnusamy V, Venkatesh V, Curley A, et al. Segmental percutaneous central venous line cultures for diagnosis of catheter-related sepsis. *Arch Dis Child Fetal Neonatal Ed* 2012;97:F273-8 doi: 10.1136/archdischild-2011-300822.
2. Ponnusamy V, Perperoglou A, Venkatesh V, et al. Skin colonisation at the catheter exit site is strongly associated with catheter colonisation and catheter-related sepsis. *Acta Paediatr* 2014;103:1233-8 doi: 10.1111/apa.12779.
3. Samarasekara SI, Booth D, Clarke P. Devastating coagulase-negative staphylococcal septicaemia in an extremely low birth weight infant. *BMJ Case Rep* 2012;DOI 10.1136/bcr-2012-007407.
4. McGovern M, Flynn L, Coyne S, et al. Does coagulase negative staphylococcal sepsis cause neurodevelopmental delay in preterm infants? *Arch Dis Child*. 2018. e-pub Oct 3. doi: 10.1136/archdischild-2018-316004.
5. Department of Health (2010). Saving Lives: reducing infection, delivering clean and safe care. High Impact Intervention No 1. Central venous catheter care bundle. Available at: https://webarchive.nationalarchives.gov.uk/2012011 (Last accessed 18th November 2018).
6. Payne V, Hall M, Prieto J, et al. Care bundles to reduce central line-associated bloodstream infections in the neonatal unit: a systematic review and meta-analysis. *Arch Dis Child Fetal Neonatal Ed* 2018;103:F422-F29 doi: 10.1136/archdischild-2017-313362.
7. Clarke P, Webber MA. Catheter sepsis and antisepsis: matters of life, death, obscurity and resistance. *Arch Dis Child Fetal Neonatal Ed* 2018;103:F94-F96 doi: 10.1136/archdischild-2017-313150.
8. Hibbard JS, Mulberry GK, Brady AR. A clinical study comparing the skin antisepsis and safety of ChloraPrep, 70% isopropyl alcohol, and 2% aqueous chlorhexidine. *J Infus Nurs* 2002;25:244-9.
9. Chaiyakunapruk N, Veenstra DL, Lipsky BA, et al. Chlorhexidine compared with povidone-iodine solution for vascular catheter-site care: a meta-analysis. *Ann Intern Med* 2002;136:792-801.
10. Pratt RJ, Pellowe CM, Wilson JA, et al. epic2: National evidence-based guidelines for preventing healthcare-associated infections in NHS hospitals in England. *J Hosp Infect* 2007;65 Suppl 1:S1-64 doi: 10.1016/S0195-6701(07)60002-4.
11. Loveday HP, Wilson JA, Pratt RJ, et al. epic3: national evidence-based guidelines for preventing healthcare-associated infections in NHS hospitals in England. *J Hosp Infect* 2014;86 Suppl 1:S1-70 doi: 10.1016/S0195-6701(13)60012-2.
12. Heron TJ, Faraday CM, Clarke P. The hidden harms of Matching Michigan. *Arch Dis Child Fetal Neonatal Ed* 2013;98:F466-7 doi: 10.1136/archdischild-2013-304378.



For peer review only - http://bmjopen.bmj.com/site/about/guidelines.xhtml

13. Garland JS, Alex CP, Mueller CD, et al. A randomized trial comparing povidone-iodine to a chlorhexidine gluconate-impregnated dressing for prevention of central venous catheter infections in neonates. *Pediatrics* 2001;107:1431-6
14. Kieran EA, O'Sullivan A, Miletin J, et al. 2% chlorhexidine-70% isopropyl alcohol versus 10% povidone-iodine for insertion site cleaning before central line insertion in preterm infants: a randomised trial. *Arch Dis Child Fetal Neonatal Ed* 2018;103:F101-F06 doi: 10.1136/archdischild-2016-312193.
15. Ponnusamy V, Venkatesh V, Clarke P. Skin antisepsis in the neonate: what should we use? *Curr Opin Infect Dis* 2014;27:244-50 doi: 10.1097/QCO.0000000000000064.
16. Biermann CD, Kribs A, Roth B, et al. Use and Cutaneous Side Effects of Skin Antiseptics in Extremely Low Birth Weight Infants - A Retrospective Survey of the German NICUs. *Klin Padiatr* 2016;228:208-12 doi: 10.1055/s-0042-104122.
17. Vivier PM, Lewander WJ, Martin HF, et al. Isopropyl alcohol intoxication in a neonate through chronic dermal exposure: a complication of a culturally-based umbilical care practice. *Pediatr Emerg Care* 1994;10:91-3.
18. Montgomery-Taylor S, Emery F, Anthony M. Eighteen months of 'Matching Michigan' at a United Kingdom Neonatal Intensive Care Unit. *Pediatr Infect Dis J* 2013;32:565-7. doi: 10.1097/INF.0b013e3182868389.
19. Stevens TP, Schulman J. Evidence-based approach to preventing central line-associated bloodstream infection in the NICU. *Acta Paediatr Suppl* 2012;101:11-6 doi: 10.1111/j.1651-2227.2011.02547.x.
20. Lund CH, Osborne JW. Validity and reliability of the neonatal skin condition score. *J Obstet Gynecol Neonatal Nurs* 2004;33:320-7.
21. Medicines and Healthcare products Regulatory Agency. Chlorhexidine solutions: reminder of the risk of chemical burns in premature infants. *Drug Saf Update* 2014;8:S3.
22. Maki DG, Weise CE, Sarafin HW. A semiquantitative culture method for identifying intravenous-catheter-related infection. *N Engl J Med* 1977;296:1305-9 doi: 10.1056/NEJM197706092962301.

For peer review only



For peer review only - http://bmjopen.bmj.com/site/about/guidelines.xhtml

| Procedure | Screening | Trial entry/ catheter insertion | Daily until 48 hours post-catheter removal | Catheter removal |
|---|---|---|---|---|
| Confirm Eligibility | √ | | | |
| Demographics | | √ | | |
| Consent | | √ | | |
| Randomisation | | √ | | |
| Open Study Pack/ catheter insertion | | √ | | |
| Skin Assessment [a] | | √ | √ | √ |
| Sepsis evaluation | | √ | √ | √ |
| Obtain blood culture if sepsis suspected [b] | | | √ | √ |
| Obtain two exit site skin swabs | | | | √ |
| Obtain proximal and tip catheter segments | | | | √ |
| SAE reporting [c] | | √ | √ | √ |
| Concomitant Medications (Antibiotics/antifungals only) | | √ | √ | √ |

a. Skin status to be recorded at baseline (prior to application of skin antiseptic), 10-30 minutes after catheter insertion, at 24 h (+/- 12 h), then daily until 48 h (+/- 12 h) after catheter removal using the modified neonatal contact dermatitis scoring system. Where catheter insertion is not achieved after IMP use, skin assessment for each anatomical region that was subject to IMP application will be recorded within 30 minutes following washing of the region with sterile water (to remove residual dried antiseptic compound), and then daily for a further 48 hours.

b. Peripheral blood culture obtained using standard aseptic technique.

c. Only adverse events which are serious will be recorded from the time of catheter insertion until 48 hours after catheter removal (or until 48 hours after antiseptic removal where catheterisation is unsuccessful). Only unforeseeable SAEs will be reported. Note that skin reactions to IMP use classed as moderate or severe should also to be notified to MHRA via the yellow card scheme in line with the specific current MHRA request for clinicians to report chlorhexidine-related skin burns in neonates.

**Figure 1: Trial procedures**

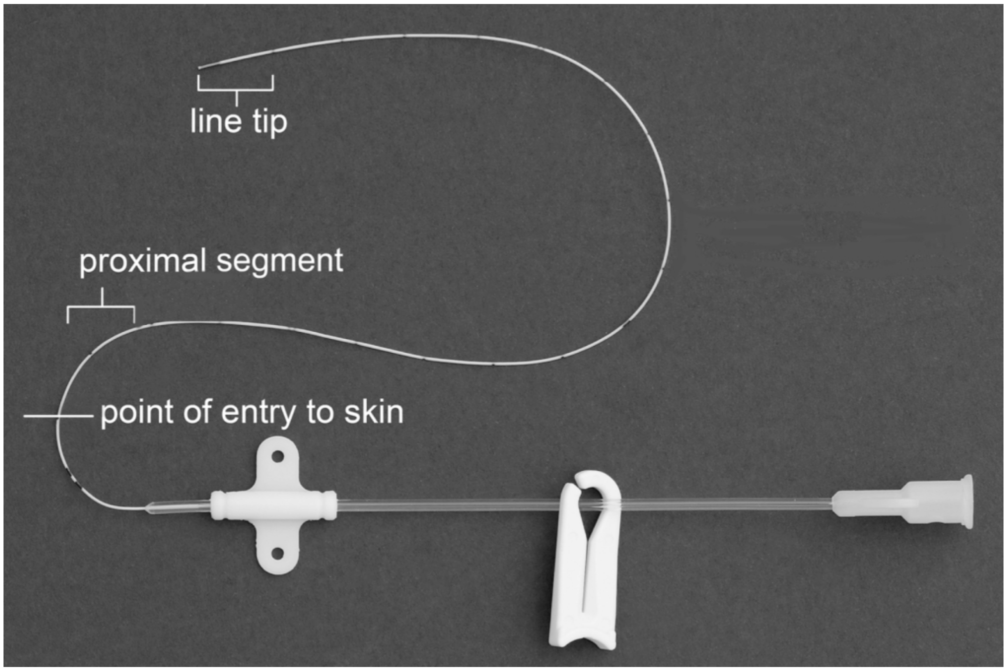


Figure 2: Catheter sections taken at the time of catheter removal

315x209mm (150 x 150 DPI)

# Reporting checklist for protocol of a clinical trial.

Based on the SPIRIT guidelines.

## Instructions to authors

Complete this checklist by entering the page numbers from your manuscript where readers will find each of the items listed below.

Your article may not currently address all the items on the checklist. Please modify your text to include the missing information. If you are certain that an item does not apply, please write "n/a" and provide a short explanation.

Upload your completed checklist as an extra file when you submit to a journal.

In your methods section, say that you used the SPIRIT reporting guidelines, and cite them as:

Chan A-W, Tetzlaff JM, Altman DG, Laupacis A, Gøtzsche PC, Krleža-Jerić K, Hróbjartsson A, Mann H, Dickersin K, Berlin J, Doré C, Parulekar W, Summerskill W, Groves T, Schulz K, Sox H, Rockhold FW, Rennie D, Moher D. SPIRIT 2013 Statement: Defining standard protocol items for clinical trials. Ann Intern Med. 2013;158(3):200-207

| | | Reporting Item | Page Number |
|---|---|---|---|
| Title | #1 | Descriptive title identifying the study design, population, interventions, and, if applicable, trial acronym | **1** |
| Trial registration | #2a | Trial identifier and registry name. If not yet registered, name of intended registry | **4** |
| Trial registration: data set | #2b | All items from the World Health Organization Trial Registration Data Set | **N/A - included in ISRCTN Trial Registration, not in this manuscript.** |
| Protocol version | #3 | Date and version identifier | **21** |
| Funding | #4 | Sources and types of financial, material, and other support | **24** |
| Roles and responsibilities: contributorship | #5a | Names, affiliations, and roles of protocol contributors | **1-2, & 24** |

For peer review only

| | | | |
|---|---|---|---|
| Roles and responsibilities: sponsor contact information | #5b | Name and contact information for the trial sponsor | **25** |
| Roles and responsibilities: sponsor and funder | #5c | Role of study sponsor and funders, if any, in study design; collection, management, analysis, and interpretation of data; writing of the report; and the decision to submit the report for publication, including whether they will have ultimate authority over any of these activities | **25** |
| Roles and responsibilities: committees | #5d | Composition, roles, and responsibilities of the coordinating centre, steering committee, endpoint adjudication committee, data management team, and other individuals or groups overseeing the trial, if applicable (see Item 21a for data monitoring committee) | **21** |
| Background and rationale | #6a | Description of research question and justification for undertaking the trial, including summary of relevant studies (published and unpublished) examining benefits and harms for each intervention | **6-8** |
| Background and rationale: choice of comparators | #6b | Explanation for choice of comparators | **7-8** |
| Objectives | #7 | Specific objectives or hypotheses | **8** |
| Trial design | #8 | Description of trial design including type of trial (eg, parallel group, crossover, factorial, single group), allocation ratio, and framework (eg, superiority, equivalence, non-inferiority, exploratory) | **9, 11** |
| Study setting | #9 | Description of study settings (eg, community clinic, academic hospital) and list of countries where data will be collected. Reference to where list of study sites can be obtained | **9** |

For peer review only

| | | | |
|---|---|---|---|
| Eligibility criteria | #10 | Inclusion and exclusion criteria for participants. If applicable, eligibility criteria for study centres and individuals who will perform the interventions (eg, surgeons, psychotherapists) | **9, 10** |
| Interventions: description | #11a | Interventions for each group with sufficient detail to allow replication, including how and when they will be administered | **11-14** |
| Interventions: modifications | #11b | Criteria for discontinuing or modifying allocated interventions for a given trial participant (eg, drug dose change in response to harms, participant request, or improving / worsening disease) | **16** |
| Interventions: adherance | #11c | Strategies to improve adherence to intervention protocols, and any procedures for monitoring adherence (eg, drug tablet return; laboratory tests) | **N/A - this is feasibility trial and adherence is one of outcomes being assessed.** |
| Interventions: concomitant care | #11d | Relevant concomitant care and interventions that are permitted or prohibited during the trial | **15** |
| Outcomes | #12 | Primary, secondary, and other outcomes, including the specific measurement variable (eg, systolic blood pressure), analysis metric (eg, change from baseline, final value, time to event), method of aggregation (eg, median, proportion), and time point for each outcome. Explanation of the clinical relevance of chosen efficacy and harm outcomes is strongly recommended | **15-16** |
| Participant timeline | #13 | Time schedule of enrolment, interventions (including any run-ins and washouts), assessments, and visits for participants. A schematic diagram is highly recommended (see Figure) | **11, Figure 1** |
| Sample size | #14 | Estimated number of participants needed to achieve study objectives and how it was determined, including clinical and statistical | **17-18** |

For peer review only

| | | | |
|---|---|---|---|
| | | assumptions supporting any sample size calculations | |
| Recruitment | #15 | Strategies for achieving adequate participant enrolment to reach target sample size | 10, 11, 17-18 |
| Allocation: sequence generation | #16a | Method of generating the allocation sequence (eg, computer-generated random numbers), and list of any factors for stratification. To reduce predictability of a random sequence, details of any planned restriction (eg, blocking) should be provided in a separate document that is unavailable to those who enrol participants or assign interventions | 11 |
| Allocation concealment mechanism | #16b | Mechanism of implementing the allocation sequence (eg, central telephone; sequentially numbered, opaque, sealed envelopes), describing any steps to conceal the sequence until interventions are assigned | 11 |
| Allocation: implementation | #16c | Who will generate the allocation sequence, who will enrol participants, and who will assign participants to interventions | 10, 11 |
| Blinding (masking) | #17a | Who will be blinded after assignment to interventions (eg, trial participants, care providers, outcome assessors, data analysts), and how | 12, 16-17 |
| Blinding (masking): emergency unblinding | #17b | If blinded, circumstances under which unblinding is permissible, and procedure for revealing a participant's allocated intervention during the trial | 16-17 |
| Data collection plan | #18a | Plans for assessment and collection of outcome, baseline, and other trial data, including any related processes to promote data quality (eg, duplicate measurements, training of assessors) and a description of study instruments (eg, questionnaires, laboratory tests) along with their reliability and validity, if | 18-19 |

For peer review only

| | | | |
|---|---|---|---|
| | | known. Reference to where data collection forms can be found, if not in the protocol | |
| Data collection plan: retention | #18b | Plans to promote participant retention and complete follow-up, including list of any outcome data to be collected for participants who discontinue or deviate from intervention protocols | **N/A - retention rates is one of the secondary outcome measures which this feasibility study is assessing** |
| Data management | #19 | Plans for data entry, coding, security, and storage, including any related processes to promote data quality (eg, double data entry; range checks for data values). Reference to where details of data management procedures can be found, if not in the protocol | **18-19** |
| Statistics: outcomes | #20a | Statistical methods for analysing primary and secondary outcomes. Reference to where other details of the statistical analysis plan can be found, if not in the protocol | **16-18** |
| Statistics: additional analyses | #20b | Methods for any additional analyses (eg, subgroup and adjusted analyses) | **19** |
| Statistics: analysis population and missing data | #20c | Definition of analysis population relating to protocol non-adherence (eg, as randomised analysis), and any statistical methods to handle missing data (eg, multiple imputation) | **N/A - this is feasibility study and rates of non completion/missing primary outcome data will be reported as outcomes.** |
| Data monitoring: formal committee | #21a | Composition of data monitoring committee (DMC); summary of its role and reporting structure; statement of whether it is independent from the sponsor and competing interests; and reference to where further details about its charter can be found, if not in the protocol. Alternatively, an explanation of why a DMC is not needed | **21** |
| Data monitoring: interim analysis | #21b | Description of any interim analyses and stopping guidelines, including who will have | **21** |

For peer review only

| | | | |
|---|---|---|---|
| | | access to these interim results and make the final decision to terminate the trial | |
| Harms | #22 | Plans for collecting, assessing, reporting, and managing solicited and spontaneously reported adverse events and other unintended effects of trial interventions or trial conduct | **20-21** |
| Auditing | #23 | Frequency and procedures for auditing trial conduct, if any, and whether the process will be independent from investigators and the sponsor | **20** |
| Research ethics approval | #24 | Plans for seeking research ethics committee / institutional review board (REC / IRB) approval | **21-22** |
| Protocol amendments | #25 | Plans for communicating important protocol modifications (eg, changes to eligibility criteria, outcomes, analyses) to relevant parties (eg, investigators, REC / IRBs, trial participants, trial registries, journals, regulators) | **N/A - there were no further changes to the protocol v3.0 dated 18 NOV 2016 during the active recruitment phase.** |
| Consent or assent | #26a | Who will obtain informed consent or assent from potential trial participants or authorised surrogates, and how (see Item 32) | **10** |
| Consent or assent: ancillary studies | #26b | Additional consent provisions for collection and use of participant data and biological specimens in ancillary studies, if applicable | **N/A** |
| Confidentiality | #27 | How personal information about potential and enrolled participants will be collected, shared, and maintained in order to protect confidentiality before, during, and after the trial | **18-19** |
| Declaration of interests | #28 | Financial and other competing interests for principal investigators for the overall trial and each study site | **25** |
| Data access | #29 | Statement of who will have access to the final trial dataset, and disclosure of contractual agreements that limit such access for investigators | **25** |

For peer review only

| | | | |
|---|---|---|---|
| Ancillary and post trial care | #30 | Provisions, if any, for ancillary and post-trial care, and for compensation to those who suffer harm from trial participation | **N/A** |
| Dissemination policy: trial results | #31a | Plans for investigators and sponsor to communicate trial results to participants, healthcare professionals, the public, and other relevant groups (eg, via publication, reporting in results databases, or other data sharing arrangements), including any publication restrictions | **21-22** |
| Dissemination policy: authorship | #31b | Authorship eligibility guidelines and any intended use of professional writers | **N/A - eligibility for authorship will follow ICMJE guidelines.** |
| Dissemination policy: reproducible research | #31c | Plans, if any, for granting public access to the full protocol, participant-level dataset, and statistical code | **21** |
| Informed consent materials | #32 | Model consent form and other related documentation given to participants and authorised surrogates | **Supplementary Appendix 1** |
| Biological specimens | #33 | Plans for collection, laboratory evaluation, and storage of biological specimens for genetic or molecular analysis in the current trial and for future use in ancillary studies, if applicable | **14-15** |

The SPIRIT checklist is distributed under the terms of the Creative Commons Attribution License CC-BY-ND 3.0. This checklist can be completed online using https://www.goodreports.org/, a tool made by the EQUATOR Network in collaboration with Penelope.ai

For peer review only

BMJ Open

# Safety and efficacy of 2% chlorhexidine gluconate aqueous versus 2% chlorhexidine gluconate in 70% isopropyl alcohol for skin disinfection prior to percutaneous central venous catheter insertion in preterm neonates: the ARCTIC randomised-controlled feasibility trial protocol

| | |
|---|---|
| Journal: | *BMJ Open* |
| Manuscript ID | bmjopen-2018-028022.R1 |
| Article Type: | Protocol |
| Date Submitted by the Author: | 26-Nov-2018 |
| Complete List of Authors: | Clarke, Paul; Norfolk and Norwich University Hospitals NHS Foundation Trust, Neonatal intensive Care Unit; University of East Anglia, Norwich Medical School<br>Craig, Jean; University of East Anglia, Research Design Service, Norwich Medical School<br>Wain, John; University of East Anglia Faculty of Medicine and Health Sciences, Norwich Medical School; Quadram Institute Biomedicine<br>Tremlett, Catherine; Norfolk and Norwich University Hospitals NHS Foundation Trust, Department of Microbiology<br>Linsell, Louise; University of Oxford, National Perinatal Epidemiology Unit, Nuffield Department of Population Health<br>Bowler, Ursula; University of Oxford, National Perinatal Epidemiology Unit, Nuffield Department of Population Health,<br>Juszczak, Ed; University of Oxford, National Perinatal Epidemiology Unit, Nuffield Department of Population Health,<br>Heath, Paul; St George's University of London, Paediatric Infectious Diseases Research Group, Infection and Immunity, |
| <b>Primary Subject Heading</b>: | Infectious diseases |
| Secondary Subject Heading: | Paediatrics |
| Keywords: | antiseptic, biocide, disinfection, central line associated bloodstream infection, catheter |
| | |

SCHOLARONE™
Manuscripts

**TITLE**

# Safety and efficacy of 2% chlorhexidine gluconate aqueous versus 2% chlorhexidine gluconate in 70% isopropyl alcohol for skin disinfection prior to percutaneous central venous catheter insertion in preterm neonates: the ARCTIC randomised-controlled feasibility trial protocol

**Authors**

Paul Clarke[1,2] (Corresponding Author)

Jean Craig[3]

John Wain[2,4]

Catherine Tremlett[5]

Louise Linsell[6]

Ursula Bowler[6]

Ed Juszczak[6]

Paul T. Heath[7]

1. Neonatal Unit, Norfolk and Norwich University Hospitals NHS Foundation Trust, Colney Lane, Norwich, Norfolk, NR4 7UY, UK.

2. Norwich Medical School, University of East Anglia, Norwich, UK

3. Research Design Service, Norwich Medical School, University of East Anglia, Norwich, UK

4. Quadram Institute Bioscience, Norwich Research Park, Norwich, UK.

5. Department of Microbiology, Norfolk and Norwich University Hospitals NHS Foundation Trust, Norwich, UK



For peer review only - http://bmjopen.bmj.com/site/about/guidelines.xhtml

For peer review only

6. National Perinatal Epidemiology Unit, Nuffield Department of Population Health, University of Oxford, Oxford OX3 7LF

7. Paediatric Infectious Diseases Research Group, Infection and Immunity, St. George's University of London, London, UK

[1.]Corresponding Author: Prof Paul Clarke paul.clarke@nnuh.nhs.uk Tel. +44 1603 286342

For peer review only

**Word count: 4238**



For peer review only - http://bmjopen.bmj.com/site/about/guidelines.xhtml

## ABSTRACT

**Introduction:** Catheter-related sepsis is one of the most dangerous complications of neonatal intensive care and is associated with significant morbidity and mortality**.** Use of catheter-care 'bundles' has reduced the incidence of catheter-related sepsis, though individual components have not been well studied. Better evidence is needed to guide selection of the most appropriate antiseptic solution for skin disinfection in preterm neonates. This study will inform the feasibility and design of the first randomised controlled trial to examine the safety and efficacy of alcohol-based versus aqueous-based chlorhexidine antiseptic formulations for skin disinfection prior to percutaneous central venous catheterisation in preterm neonates. The antiseptics to be compared are 2% CHG aqueous and 2% CHG in 70% isopropyl alcohol.

**Methods and Analysis:** The Antiseptic Randomised Controlled Trial for Insertion of Catheters (ARCTIC) is a two-centre randomised-controlled feasibility trial. At least 100 preterm infants born at <34 weeks' gestation and due to undergo percutaneous insertion of a central venous catheter will be randomly allocated to receive prior skin disinfection with one of the two antiseptic solutions. Outcomes include: i) recruitment and retention rates; ii) completeness of data collection; iii) numbers of enrolled infants meeting case definitions for definite catheter-related sepsis, catheter-associated sepsis, and catheter colonisation; and iv) safety outcomes of skin morbidity scores recorded daily from catheter insertion until 48 hours post removal. The key feasibility metrics will be reported as proportions with 95% confidence intervals. Estimated prevalence of catheter colonisation will allow calculation of sample size for the large-scale trial. The data will inform whether it will be feasible to progress to a large-scale trial.




For peer review only - http://bmjopen.bmj.com/site/about/guidelines.xhtml

**Ethics and dissemination:** ARCTIC has been approved by the National Health Service Health Research Authority National Research Ethics Service Committee East of England (Cambridge South) (IRAS ID 163868), was adopted onto the National Institute of Health Research Clinical Research Network portfolio (CPMS ID 19899), and is registered with an International Standard Randomised Control Trials Number (ISRCTN: 82571474) and European Clinical Trials Database (EudraCT) number 2015-000874-36. Dissemination plans include presentations at scientific conferences, scientific publications, and sharing of the findings with parents via the support of Bliss baby charity.

**Registration details:** Trial registration numbers ISRCTN82571474; EudraCT No. 2015-000874-36.

**Keywords**

Antiseptic; disinfection; sepsis; central line associated bloodstream infection; trial

**Article Summary**

**Strengths and limitations of this study**

- The ARCTIC study will be one of only very few randomised controlled trials of skin antiseptics in preterm neonates and the first to compare aqueous 2% chlorhexidine gluconate versus 70% isopropyl alcohol-based 2% chlorhexidine gluconate for cutaneous disinfection prior to central venous catheterisation
- The trial will collect rigorous, prospective safety data following antiseptic application through daily skin safety assessments using a validated neonatal skin scoring tool

For peer review only

- This will be the first study in neonates to undertake molecular typing of isolates to verify that skin-colonising and blood-cultured organisms match catheter-colonising organisms to a species level in babies with suspected sepsis, thus allowing definitive proof of catheter-related sepsis
- Catheter colonisation will be used as a proxy for catheter sepsis, and the target sample size is based upon an anticipated incidence of catheter colonisation of 20% in the reference antiseptic group, estimated with a 95% Confidence Interval of 11% to 31%, and is not powered to detect differences in clinical outcomes
- This trial will show whether a future large-scale multicentre randomised controlled non-inferiority trial of the same antiseptics is feasible and will determine the sample size required for such a trial

For peer review only



For peer review only - http://bmjopen.bmj.com/site/about/guidelines.xhtml

## Introduction

Percutaneously-inserted central venous catheters (PCVCs) are inserted daily in neonatal intensive care units (NICUs) across the world to deliver hyperosmolar parenteral nutrition solutions to preterm neonates. PCVCs may remain in situ for weeks[1], but their presence entails a major risk for bloodstream infection. In a previous study, 32% of inserted PCVCs were colonised with potentially-pathogenic bacteria at the point of removal, and 8% overall were associated with definite catheter-related sepsis (CRS).[1] Extraluminal colonisation is the main route of catheter colonisation in short-term CVCs: skin bacteria traverse the catheter insertion site onto the catheter, colonise the line, and act as focus for CRS.[2] In one study the presence of skin bacteria at the catheter exit site was associated with an 8-fold increased risk of catheter colonisation and a 10-fold increased risk of CRS caused by the same organism.[2]

For preterm babies in the NICU, CRS is a dangerous complication associated with significant morbidity and mortality**.** Sepsis increases the duration of intensive care and hospitalisation, need for antibiotics, and risks for adverse neurodevelopmental outcome**.** Coagulase-negative Staphylococcal infections cause the majority of CRS in the NICU (80-90%), may be life-threatening, and can cause permanent lifelong injury and disability in survivors, including cerebral palsy.[3,4]

Reduction of CRS has been a major goal of the National Health Service for the past decade.[5] Catheter-care ‘bundles’, guidelines incorporating collected good practices for catheter insertion and maintenance, have successfully reduced the incidence of catheter colonisation and CRS in the NICU,[6] though await universal adoption.[7]



For peer review only - http://bmjopen.bmj.com/site/about/guidelines.xhtml

The individual components of catheter-care bundles have not been well studied in randomised-controlled trials (RCTs). Adequate skin disinfection of the catheter insertion site is arguably the most important component of catheter-care bundles to prevent catheter colonisation and CRS. Optimal skin preparation will abolish or significantly reduce numbers of skin organisms, so limiting risks of residual skin colonisation by bacteria that may then colonise a PCVC and cause CRS.

Studies in adults, including meta-analysis, show that alcohol-based antiseptics are superior for topical antisepsis,[8,9] and UK national evidence-based guidelines recommend use of 2% chlorhexidine (CHG) in 70% isopropyl alcohol for skin antisepsis in adults and older children. However there is no guidance for preferred antiseptic in infants, including preterm infants, due to the lack of evidence and specific safety concerns in this population.[10,11] The best antiseptic to use for preterm babies is still unknown, and multiple different antiseptics, combinations, and concentrations are presently being used in UK NICUs; approximately half using a 2% concentration of CHG and 60% an isopropyl alcohol-containing CHG formulation.[12] For preterm neonates, there is no Cochrane review comparing skin antiseptics for cutaneous disinfection prior to PCVC insertion, and only two RCTs have compared topical antiseptics for PCVC insertion.[13,14]

There are risks associated with antiseptic use peculiar to preterm infants. Their thin skin is vulnerable to chemical injury and absorption. Chemical skin burns have been described with all the currently-used topical antiseptics, including both aqueous and alcohol-containing CHG formulations, and iodine solutions,[15] as well as with octenidine.[16] Topical alcohol use may also increase the risk of systemic chemical absorption.[17]



For peer review only - http://bmjopen.bmj.com/site/about/guidelines.xhtml

There are no published RCTs that have examined the safety and efficacy of alcohol-based versus aqueous CHG formulations for neonatal antisepsis. This feasibility study aims to inform the safety and assist the design and planning of a future large-scale multicentre RCT that will examine whether 2% CHG aqueous is non-inferior in antiseptic efficacy compared to 2%CHG-70%IPA for skin disinfection prior to PCVC insertion in preterm neonates. An aqueous CHG is likely to have fewer side effects than an alcohol-based CHG, and would therefore be preferable if found to be non-inferior in terms of antisepsis.

**OBJECTIVES**

To determine the proportion of babies in the 2%CHG-70%IPA group with colonisation of at least one of the two catheter segments taken at catheter removal. Catheter colonisation will be the primary outcome in the full-scale trial because it is a valid surrogate for CRS;[2] to determine factors affecting recruitment and process outcomes that will help refine the design of the large-scale trial; also to estimate numbers of enrolled infants who have definite CRS and numbers with catheter-associated sepsis, determine suitability and completeness of data collection methods, and describe any skin morbidity occurring in trial participants related to use of the study antiseptics.



For peer review only - http://bmjopen.bmj.com/site/about/guidelines.xhtml

## Methods and analysis:

### Study design

A feasibility, masked randomised controlled trial of investigational medical products (IMPs). Preterm infants born at <34 weeks' gestation who are due to undergo planned insertion of a PCVC will be randomised to receive one of two topical disinfection agents for skin antisepsis: 2% chlorhexidine gluconate aqueous (2%CHG-aqueous), or 2% chlorhexidine gluconate in 70% isopropyl alcohol (2%CHG-70%IPA).

### Study Setting

Two tertiary-level neonatal units in the UK, Norfolk and Norwich University Hospital and Medway Maritime Hospital, which each cater to a total of 5000-6000 deliveries per year.

### Eligibility

#### Inclusion criteria

- Preterm infants born at <34 weeks' gestation
- Requiring routine insertion of a PCVC for parenteral nutrition
- No new episode of suspected sepsis with commencement of antibiotics occurring within the 48 hours preceding planned catheter insertion
- No other indwelling PCVC already in situ

#### Exclusion criteria

- No realistic prospect of survival in the short term
- Life-threatening congenital abnormality
- Underlying skin condition



For peer review only - http://bmjopen.bmj.com/site/about/guidelines.xhtml

- Another indwelling PCVC already in situ or previously enrolled into the study
- Positive blood culture (BC) within the past 7 days without a subsequent negative BC result
- Antibiotic treatment commenced for suspected sepsis within the preceding 48 hours

**Key Definitions**

*Definite catheter-related sepsis* (CRS): a peripheral BC plus any catheter segment (i.e. one of the ~1cm long proximal or tip catheter portions) positive with the same organism, based on bacterial culture, antibiotic sensitivity and molecular typing, from a neonate who had an indwelling PCVC and clinical signs of sepsis but no other focus of sepsis

*Catheter colonisation*: a catheter that at the time of removal has either one or both segments that are culture positive.

*Catheter-associated sepsis*: a baby with clinical signs of sepsis and an accompanying positive BC in the period between catheter insertion and 48 hours post removal but who has no other focus of sepsis and in whom both catheter segment cultures are negative

**Recruitment**

Preterm babies potentially suitable for the trial will be identified by the clinical healthcare teams. Parents of such infants will be approached for consent by the research team or delegated suitably-qualified member of the clinical healthcare team trained in study procedures and Good Clinical Practice (GCP). A written parent information sheet that forms part of the parental Informed Consent Form will be provided to help explain the study (Supplemental File). Written maternal consent will be obtained and countersigned by the person who obtained informed consent (principal investigator [PI], or appropriately qualified healthcare professional with delegated authority).



For peer review only - http://bmjopen.bmj.com/site/about/guidelines.xhtml

## Randomisation

Following consent, randomisation to either 2%CHG-70%IPA antiseptic or 2%CHG-aqeuous antiseptic will take place as close as possible to the time of planned catheter insertion. Randomisation will be managed via a secure web-based facility hosted by the National Perinatal Epidemiology Unit Clinical Trials Unit (CTU) and will use a 3:1 allocation ratio in favour of the 2%CHG-70%IPA antiseptic group, the group that will inform the power calculation for the large-scale trial. Groups will be stratified by birth gestation (<28 weeks; 28 weeks+0 days to 33 weeks+6 days) and by centre. Treatment allocation will be masked such that the allocation will not be known by clinicians, the baby's family, laboratory staff, or trial outcome assessors.

## Interventions

The Trial procedures are summarised in **Figure 1.**

## Skin Disinfection at Catheter Insertion

Study packs stored in a locked, secure, temperature-monitored cupboard will contain two bottles of the same allocated antiseptic IMP each labelled with the same identifying code. One bottle will be opened at catheter insertion, the other will be retained for use at catheter removal. The disinfection procedure requires sparing application of allocated antiseptic solution for 10-20 seconds from sterile gauze to the skin site area selected for catheterisation. Instructions are: use only the minimal volume of antiseptic necessary for skin coverage, avoid any pooling of antiseptic, ensure that any excess solution and any



For peer review only - http://bmjopen.bmj.com/site/about/guidelines.xhtml

soaked drapes or gowns are removed to avoid any prolonged contact with the skin, and allow the disinfected area to air dry completely (for ≥30 seconds) before proceeding with catheter insertion. Excepting in the case of failed catheterisation, saline or water must not be used to wipe the disinfected skin area following application of antiseptic solution.

**Catheter Insertion**

Catheter insertion will be standardised, using a working guideline common to both participating centres that requires strict aseptic technique and encompasses established good clinical practices for PCVC insertion and care adopted from catheter-care bundles.[5, ,18,19] The decision to insert a PCVC and choice of catheter (Premicath 1Fr/28G or Epicutaneo-Cava catheter 2Fr/24G: both Vygon UK Ltd, Cirencester, UK) is at the discretion of the attending clinical team. All personnel involved will be trained in catheter insertion and maintenance procedures. An insertion checklist will be completed for all catheterisations and a disposable face mask will be worn by the operator inserting the catheter for asepsis purposes and also to minimise the risk of possible unblinding from any smell of alcohol.

**Assessment of Skin Condition**

Skin status will be recorded using a validated neonatal contact dermatitis scoring system, the Neonatal Skin Condition Score,[20] with minor modification. Assessments will be undertaken by a nurse trained in use of the scoring system and will be recorded at baseline (prior to application of antiseptic), within 10-30 minutes after catheter insertion, and then daily until 48 hours following catheter removal, or daily for 48 hours after antiseptic application in cases where catheterisation is unsuccessful. Serious chemical skin burns



For peer review only - http://bmjopen.bmj.com/site/about/guidelines.xhtml

adjudged by a PI or delegate to be severe or moderately severe, will be notified to the Medicines and Healthcare Products Regulatory Agency (MHRA).[21]

**Catheter Removal and obtainment of Study specimens**

Catheters are usually removed when no longer required though sometimes removal is warranted earlier than intended because of complications, including suspected sepsis. If a catheter is being removed from a baby with suspected sepsis then a concurrent peripheral BC will be obtained as per routine clinical practice. The decision for catheter removal for enrolled babies lies with the attendant clinical team.

The following samples will be obtained at the time of catheter removal for microbiological analysis:

i) Two exit site skin swabs taken *before* catheter removal: the first after removing the transparent covering dressings but prior to skin disinfection; the second taken post new disinfection of the insertion site, once the antiseptic has dried. This disinfection procedure is intended to limit the risk of catheter contamination by residual skin organisms during the removal of the catheter and will use the same allocated antiseptic as was used for catheter insertion. Both specimens will be obtained by rolling the swab tip several times across the skin of the catheter insertion site, over an area within <0.5 cm radius of the insertion site but also including the actual puncture site.

ii) Two approximately 1 cm long catheter segments (proximal and tip) *after* catheter removal. Proximal catheter segments have higher colonisation rates than tips,[1] therefore microbiological analysis of both catheter segments rather than the tip alone may improve the diagnostic yield of catheter colonisation.



For peer review only - http://bmjopen.bmj.com/site/about/guidelines.xhtml

Catheter removal requires two trained persons and care to avoid cross-contamination between segments while sectioning the catheter. Two separate sets of sterile forceps, two pairs of sterile scissors, and two sterile pre-labelled universal containers are required. Before removing the catheter, the subcutaneous insertion length will be noted from the external catheter markings. The catheter will be removed onto a sterile paper towel field then sectioned using separate pairs of sterile scissors to obtain two ~1 cm-long formerly subcutaneous catheter segments: i) tip, and ii) a proximal segment, taken approximately 1-2 cm distal to the point of skin entry (**figure 2**). The individual segments will be placed into separate appropriately-labelled sterile universal containers using separate pairs of sterile forceps.

**Microbiology**

The catheter segments and skin swabs will be submitted to the local microbiology laboratory for routine culture and antibiotic sensitivities. BCs sent from babies with suspected sepsis at the time of catheter removal will undergo standard culture methods. Bacterial growths from skin swab cultures will be assessed using a semi-quantitative method.[22] All laboratory staff will be blinded to antiseptic allocation. Isolates from culture-positive skin swabs, blood, and catheter segmental cultures will be retained for molecular typing. Initial identification of organisms will be done by Mass Spectrometry. Those giving similar patterns will be analysed using Next Generation sequencing using a multiplexed approach on the Illumina MiSeq. Molecular typing of paired blood and catheter isolates from the same baby will allow confirmation that isolates are identical to a species level, for definitive diagnosis of definite CRS. While few post-disinfection skin swabs are



For peer review only - http://bmjopen.bmj.com/site/about/guidelines.xhtml

expected to be positive, molecular typing of skin swab isolates will be done for any babies with colonised catheters: isolation of paired identical species could indicate possible catheter contamination by skin organisms during catheter removal. Babies with positive BCs will be managed according to local clinical guidelines; involvement in this trial will not dictate or influence clinical antibiotic prescriptions.

**Outcome measures**

I. Proportion of babies in the 2%CHG-70%IPA arm with catheter colonisation as determined by positive bacterial culture from one or both of the catheter segments taken at catheter removal (primary outcome)

II. Rates of recruitment and retention to the study, and the collection of views of parents and clinicians on factors affecting recruitment and retention

III. Proportion of infants with positive exit-site skin swabs at catheter removal

IV. Number and type of catheter segments culture positive at removal

V. Bacterial species (typed via molecular methods) of isolates identified on positive BC, exit-site skin swabs, and catheter segments

VI. Proportion of infants undergoing an infection screen in the period between catheter insertion and 48 hours post catheter removal that meets case definition for definite CRS

VII. Proportion of infants with positive blood culture from any infection screen in the period between catheter insertion and 48 hours post-catheter removal that meets definition for catheter-associated sepsis

VIII. Rate of CRS per 1000 PCVC days

IX. Rate of catheter-associated sepsis per 1000 PCVC days



For peer review only - http://bmjopen.bmj.com/site/about/guidelines.xhtml

For peer review only

X. Proportion of infants completing study with complete data for primary outcome and proportions of infants with missing data collection forms

XI. Daily skin morbidity scores in the period between catheter insertion and 48 hours post-catheter removal, and in the period between antiseptic application and 48 hours post antiseptic application where catheterisation was unsuccessful.

**Supportive care of participants**

The clinical management of babies enrolled in the study will follow standard local practices. For the purposes of this feasibility study, if CRS is suspected the ideal will be catheter removal at that time. However, it is recognised that a pragmatic approach is sometimes needed, especially for very premature babies in whom catheter replacement may be difficult and challenging. Efforts will be made to minimise local differences in treatment practices between sites through training.

**Discontinuation of Trial intervention**

The trial intervention will be stopped on parental request, or if the baby develops serious adverse skin damage that, in the opinion of the responsible PI, was caused by the IMP. Thus, if any baby has a clinically-significant chemical skin burn following IMP application at catheter insertion then the allocated antiseptic will be withheld from use for skin disinfection at catheter removal. In such instance skin swab and catheter sections will still be obtained as per removal procedure and the protocol deviation will be recorded.

**Blinding and unblinding**

The antiseptic IMPs will be manufactured by a MHRA-accredited Specialist Pharmacy



For peer review only - http://bmjopen.bmj.com/site/about/guidelines.xhtml

Manufacturing Unit compliant with Good Manufacturing Practices. The IMP will be supplied in bottles and both products will be coloured pink (using carmoisine) and visually indistinguishable. To maintain blinding, each baby will be issued a unique allocation number corresponding to the study pack number. Emergency unblinding for valid medical or safety reasons is via the randomisation website using a single-use access code provided in a sealed envelope in the Investigator Site File.

**Sample size**

Our previous study found that 32% of PCVCs had a colonised tip and/or proximal segment at removal after using much weaker concentration (0.015%-0.05%) CHG solutions and the definite CRS rate was 6.8 per 1000 catheter days.[1] In comparison, a UK NICU that routinely used 2%CHG-70%IPA for PCVC insertions reported a 34% lower CRS rate (4.5 per 1000 catheter days).[18] Presuming that alcohol-based 2%CHG is the major factor of benefit in catheter care, by extrapolation we might reasonably expect to see a 34% reduction in extraluminal catheter colonisation rates by using 2%CHG-70%IPA solution. Thus we estimate a catheter colonisation rate of approximately 20% (0.66 x 32% = 21%) may be achieved with 2%CHG-70%IPA solution. A 3:1 allocation ratio in favour of the reference 2%CHG-70%IPA group requires a target sample size of approximately 93 babies with successfully inserted (and removed) catheters (i.e. n=70 in the reference group) to estimate the critical parameters for a future, large-scale trial with an adequate degree of precision. With this target sample size, the anticipated incidence of the primary outcome in the reference group of 20% will be estimated with a 95% Confidence Interval of 11% to 31%. With a sample size of 93 babies with successfully inserted catheters, the anticipated recruitment/uptake rate of 75%[13] will be estimated with a 95% Confidence Interval of 0.65 to 0.83. A sample size of



For peer review only - http://bmjopen.bmj.com/site/about/guidelines.xhtml

approximately 93 babies having catheters successfully inserted/removed will require parents of at least 124 eligible babies to be approached.

**Data management and analysis**

Outcome data include routinely recorded clinical information obtainable from clinical and local microbiological laboratory records. Data verifying species of catheter colonisation will be collected following further analysis of positive isolates by molecular typing. Data will be collected using study-specific data collection forms for: Trial Entry and Randomisation, Main Outcome Data (catheter insertion, skin condition assessment, sepsis evaluation, and antimicrobial therapy), Catheter Removal, Microbiology Data, Unsuccessful Catheterisation Episodes, Discontinuation of Intervention, Withdrawal, and Foreseeable Serious Adverse Events (SAE). In addition, information will be collected and reported to the sponsor using the sponsor's SAE report Form and Incident Form, to report any deviation from the protocol, trial-specific procedures, or GCP.

For peer review only

Data collection will proceed from randomisation until 48 hours post catheterisation for successfully inserted catheters, or until 48 hours after last antiseptic application for unsuccessful catheterisation. If a baby is discharged from its recruiting centre before study completion, to try to achieve complete follow-up safety data, the research team will contact the receiving clinical nursing team to request routine daily documentation regarding status of catheter insertion site skin and details of any new clinical sepsis events until 48 hours post catheter removal.

All data will be collected, transferred, and stored in compliance with GCP and current Data



For peer review only - http://bmjopen.bmj.com/site/about/guidelines.xhtml

Protection legislation. The trial co-ordinating centre (Norwich) will hold the main administrative database for the trial. Data acquired by the enrolling units will initially be recorded onto paper data collection forms, followed by entry into an OpenClinica database (OpenClinica, Waltham, USA) administered by the National Perinatal Epidemiology Unit (NPEU). Access to this database will be via a web browser and restricted to authorised users. The database has been tested and validated prior to use. All data collection, transfer, and storage will comply with GCP and Data Protection legislation.

**Statistical Analysis Plan**

A statistical analysis plan for proposed analysis and presentation of the results of the trial will be drawn up by the delegated CTU medical statistician. Drafts will be reviewed by CTU personnel, by the CI, and by the chair of the TSC and a final version will be approved prior to the end of recruitment. Any deviations from the plan will be described and justified in the final report. Analysis will be carried out by an appropriately qualified and experienced statistician, who will ensure the integrity of the data during their processing.

**Site Training**

Each recruiting centre is staffed by a local research nurse dedicated to support the study. Initiation visits at each participating neonatal unit will be performed by the CI and study Research Nurse, also attended by the sponsor's representative. Training in study-specific procedures and in awareness of the principles of GCP will be provided for nursing and medical staff in each site by the local PI and research nurses, who will also help maintain training and delegation logs.



For peer review only - http://bmjopen.bmj.com/site/about/guidelines.xhtml

**Monitoring**

The sponsor's nominated representatives will undertake monitoring visits during the course of the study at each recruiting site to check for completeness and quality of data collection and adherence to the study protocol and reporting requirements. A monitoring plan is in place to determine the frequency and scope of site monitoring based on continuing risk review. Face-to-face monitoring visits will initially be undertaken within the first 6 months and the frequency and mode of ongoing site monitoring will be revised following assessment of recruitment rates, number of data queries, and safety/incident reports.

For peer review only

**Pharmacovigilance**

Safety of participants will be assessed continuously from randomisation until 48 hours post catheter removal. The frequency of adverse events and SAEs as defined by The International Conference on Harmonisation and that would normally require reporting within a clinical trial is expected to be high in this population. In accordance with regulatory guidance which allows for exceptions in such circumstances, a modified reporting plan was approved by the research ethics committee and by the MHRA. This plan exempted the need for routine reporting of pre-specified SAEs that are a foreseeable occurrence in preterm babies, unless considered causally related to IMP or trial procedures. Unforeseeable SAEs will be reported on the sponsor's SAE form. The relationship of each adverse event to the trial medication will be determined by a medically qualified individual. All reportable SAEs with causality assessed as 'possibly', 'probably', or 'definitely', will be considered as related to IMP. All SAEs assigned by the PI or delegate (or following sponsor/CI review) as suspected to be related to IMP *and* unexpected will be classified as suspected unexpected serious adverse reactions and subject to expedited reporting to the MHRA.



For peer review only - http://bmjopen.bmj.com/site/about/guidelines.xhtml

**Data and safety monitoring**

The Data Monitoring Committee (DMC) is responsible for safeguarding the interests of the trial participants and making recommendations to the Trial Steering Committee (TSC). The ARCTIC DMC roles, responsibilities, and operating procedures are defined in the ARCTIC DMC Charter. It is composed of three independent multidisciplinary experts who are not involved in the conduct of the trial in any way. They met prior to the initiation of enrolment and determined a plan to review the protocol, compliance, safety, and outcome data after 50 babies had been recruited. The TSC is composed of 8 independent members and has a Charter defining members' roles and responsibilities. Its Chair and the majority of the TSC membership are independent of the trial. The TSC provides the overall supervision, monitors progress and conduct of the trial, and advises on its scientific credibility. The TSC will consider and act, as appropriate, upon any recommendations of the DMC and carries ultimate responsibility for deciding whether the trial needs to be stopped on grounds of safety or efficacy. The TSC will report on trial progress to the trial funder.

**Patient and public involvement**

The study proposal benefitted from extensive patient and public involvement during its development. Advice received included regarding content of the Parental Informed Consent Form and aspects of protocol design. Input was received from Bliss baby charity (www.bliss.org.uk), by PPIRes (http://nspccro.nihr.ac.uk/public-and-patient-involvement-in-research), and by a consumer member representative of the Neonatal Clinical Specialty Group of the Medicines for Children Research Network. We also consulted with a local parent support group and with parents of babies who had suffered CRS. Two lay members



For peer review only - http://bmjopen.bmj.com/site/about/guidelines.xhtml

are involved in trial management as members of the TSC, and will be involved in dissemination of findings.

**Ethics and dissemination**

The trial received approval from the National Health Service Health Research Authority National Research Ethics Service Committee East of England (Cambridge South) (IRAS ID 163868). Clinical trial authorisation was granted by the MHRA (REF: 13630/0009/001-0001). Written approvals were received from individual hospital sites prior to recruitment. The Trial commenced recruitment under protocol version 3.0, dated 18th November 2016; full protocol is available at: https://www.npeu.ox.ac.uk/arctic. The investigator or a suitably qualified person designated by the local PI will obtain written informed consent from the patient's parent/legally-accepted representative before any trial-specific activity is performed. The CI will ensure that the trial is conducted in accordance with the principles of the Declaration of Helsinki and in conformity with GCP. The trial's findings will be presented at national and international scientific meetings and conferences and will be published in an open access peer-reviewed journal.

**Conclusions**

Recruitment to ARCTIC commenced in March 2017 and the projected overall trial end date is 31/03/2019. It is hoped that the findings of this feasibility study will pave the way for the definitive large-scale efficacy/safety study. The anticipated large-scale study will be a multi-centre non-inferiority RCT of the same two antiseptics for skin disinfection prior to PCVC insertion in preterm neonates. Primary outcome will be catheter colonisation as determined



For peer review only - http://bmjopen.bmj.com/site/about/guidelines.xhtml

by culture of one or more catheter segments taken at catheter removal. National evidence-based guidelines for preventing healthcare-associated infections in the NHS, ('epic'), commissioned by the Department of Health, were published in 2001, 2007, and 2014 to incorporate new research evidence.[10,11] Due to the lack of previous quality RCTs in the preterm population, no previous guidelines included advice or recommendations on antiseptics specific for preterm neonates. We anticipate that the findings from this research will be incorporated into a future version of the epic guidelines.

For peer review only



For peer review only - http://bmjopen.bmj.com/site/about/guidelines.xhtml

**Figure Legends:**

**Figure 1:** Trial procedures

**Figure 2:** Picture showing catheter sections taken at catheter removal

**Author Contributions:**

PC and PTH conceived the idea for this study. PC designed the study, wrote the protocol, and is Chief Investigator responsible for all aspects of the study including preparation and submission of the grant application, application for Clinical Trial Authorisation from the Licensing Authority (MHRA), securing funding, project management, data collection and preparation of the manuscript for publication. JC helped design the study and contributed to manuscript revision. PTH, JW, CT, LL, UB, and EJ helped design and refine the study, developed the protocol, and contributed to manuscript revision. LL and EJ in addition provided statistical and methodological expertise, and UB helped with the logistical aspects of the trial involving the trials unit. All authors approved the final version of the manuscript.

**Acknowledgments:** We thank Amy Nichols (research nurse) and Tracy Oliver (Study coordinator) at the Norwich site; Drs Aung Soe (PI), Dr Santosh Pattnayak (Co-Investigator) and Helen Harizaj (research nurse) at Medway site. The CI is grateful to Nigel Lambert and Susan Stirling for helpful early input into study design, and Andy King, Christopher Partlett, and Kayleigh Stanbury of NPEU CTU for help with database, programming, statistical support and trial management. We also sincerely thank Professor Kate Costeloe, TSC chair, and Dr Mark Turner, DMC chair, and the membership of these committees. PC wishes to acknowledge the support and help of Lisa Chalkley, Julie Dawson, Basia Brown, and Francesca Dockerty for the sponsor.



For peer review only - http://bmjopen.bmj.com/site/about/guidelines.xhtml

**Funding** The ARCTIC Trial is funded by the National Institute for Health Research Research for Patient Benefit Programme (Project ref: PB-PG-1013-32076).

**Sponsor** The Norfolk and Norwich University Hospitals NHS Foundation Trust is the sponsor of this trial. Sponsor contact details: R+D Manager, Research and Development Office, Level 3 East Block, Norfolk and Norwich University Hospitals NHS Foundation Trust, Colney Lane, Norwich, Norfolk, NR4 7UY. Tel. 01603 288437; Fax. 01603 289800; Email: rdoffice@nnuh.nhs.uk

**Competing interests** None declared.

**Disclaimer:** The Trial sponsor and funder (NIHR) had no role in trial design, and will have no role in gathering of data, access to data, interpretation of data, preparation of the manuscript, or decision to publish the results. The views expressed in this publication are those of the authors and not necessarily those of the NHS, NIHR or the Department of Health.

**Patient consent** Written maternal consent obtained for all participants.

**Ethics approval**: NHS Health Research Authority National Research Ethics Service Committee East of England (Cambridge South)

**Provenance and peer review:** Not commissioned; peer reviewed for ethical and funding approval prior to submission.

**Data sharing statement** Data collection is underway. The data supporting the findings of this study will be available in due course from the Chief Investigator upon reasonable request and with the permission of NPEU/sponsor.

For peer review only



For peer review only - http://bmjopen.bmj.com/site/about/guidelines.xhtml

**Open Access** This is an Open Access article distributed in accordance with the terms of the Creative Commons Attribution (CC BY 4.0) license, which permits others to distribute, remix, adapt and build upon this work, for commercial use, provided the original work is properly cited. See: http://creativecommons.org/licenses/by/4.0/

## REFERENCES

1. Ponnusamy V, Venkatesh V, Curley A, et al. Segmental percutaneous central venous line cultures for diagnosis of catheter-related sepsis. *Arch Dis Child Fetal Neonatal Ed* 2012;97:F273-8 doi: 10.1136/archdischild-2011-300822.
2. Ponnusamy V, Perperoglou A, Venkatesh V, et al. Skin colonisation at the catheter exit site is strongly associated with catheter colonisation and catheter-related sepsis. *Acta Paediatr* 2014;103:1233-8 doi: 10.1111/apa.12779.
3. Samarasekara SI, Booth D, Clarke P. Devastating coagulase-negative staphylococcal septicaemia in an extremely low birth weight infant. *BMJ Case Rep* 2012;DOI 10.1136/bcr-2012-007407.
4. McGovern M, Flynn L, Coyne S, et al. Does coagulase negative staphylococcal sepsis cause neurodevelopmental delay in preterm infants? *Arch Dis Child*. 2018. e-pub Oct 3. doi: 10.1136/archdischild-2018-316004.
5. Department of Health (2010). Saving Lives: reducing infection, delivering clean and safe care. High Impact Intervention No 1. Central venous catheter care bundle. Available at: https://webarchive.nationalarchives.gov.uk/2012011 (Last accessed 18th November 2018).
6. Payne V, Hall M, Prieto J, et al. Care bundles to reduce central line-associated bloodstream infections in the neonatal unit: a systematic review and meta-analysis. *Arch Dis Child Fetal Neonatal Ed* 2018;103:F422-F29 doi: 10.1136/archdischild-2017-313362.
7. Clarke P, Webber MA. Catheter sepsis and antisepsis: matters of life, death, obscurity and resistance. *Arch Dis Child Fetal Neonatal Ed* 2018;103:F94-F96 doi: 10.1136/archdischild-2017-313150.
8. Hibbard JS, Mulberry GK, Brady AR. A clinical study comparing the skin antisepsis and safety of ChloraPrep, 70% isopropyl alcohol, and 2% aqueous chlorhexidine. *J Infus Nurs* 2002;25:244-9.
9. Chaiyakunapruk N, Veenstra DL, Lipsky BA, et al. Chlorhexidine compared with povidone-iodine solution for vascular catheter-site care: a meta-analysis. *Ann Intern Med* 2002;136:792-801.
10. Pratt RJ, Pellowe CM, Wilson JA, et al. epic2: National evidence-based guidelines for preventing healthcare-associated infections in NHS hospitals in England. *J Hosp Infect* 2007;65 Suppl 1:S1-64 doi: 10.1016/S0195-6701(07)60002-4.
11. Loveday HP, Wilson JA, Pratt RJ, et al. epic3: national evidence-based guidelines for preventing healthcare-associated infections in NHS hospitals in England. *J Hosp Infect* 2014;86 Suppl 1:S1-70 doi: 10.1016/S0195-6701(13)60012-2.



For peer review only - http://bmjopen.bmj.com/site/about/guidelines.xhtml

12. Heron TJ, Faraday CM, Clarke P. The hidden harms of Matching Michigan. *Arch Dis Child Fetal Neonatal Ed* 2013;98:F466-7 doi: 10.1136/archdischild-2013-304378.
13. Garland JS, Alex CP, Mueller CD, et al. A randomized trial comparing povidone-iodine to a chlorhexidine gluconate-impregnated dressing for prevention of central venous catheter infections in neonates. *Pediatrics* 2001;107:1431-6
14. Kieran EA, O'Sullivan A, Miletin J, et al. 2% chlorhexidine-70% isopropyl alcohol versus 10% povidone-iodine for insertion site cleaning before central line insertion in preterm infants: a randomised trial. *Arch Dis Child Fetal Neonatal Ed* 2018;103:F101-F06 doi: 10.1136/archdischild-2016-312193.
15. Ponnusamy V, Venkatesh V, Clarke P. Skin antisepsis in the neonate: what should we use? *Curr Opin Infect Dis* 2014;27:244-50 doi: 10.1097/QCO.0000000000000064.
16. Biermann CD, Kribs A, Roth B, et al. Use and Cutaneous Side Effects of Skin Antiseptics in Extremely Low Birth Weight Infants - A Retrospective Survey of the German NICUs. *Klin Padiatr* 2016;228:208-12 doi: 10.1055/s-0042-104122.
17. Vivier PM, Lewander WJ, Martin HF, et al. Isopropyl alcohol intoxication in a neonate through chronic dermal exposure: a complication of a culturally-based umbilical care practice. *Pediatr Emerg Care* 1994;10:91-3.
18. Montgomery-Taylor S, Emery F, Anthony M. Eighteen months of 'Matching Michigan' at a United Kingdom Neonatal Intensive Care Unit. *Pediatr Infect Dis J* 2013;32:565-7. doi: 10.1097/INF.0b013e3182868389.
19. Stevens TP, Schulman J. Evidence-based approach to preventing central line-associated bloodstream infection in the NICU. *Acta Paediatr Suppl* 2012;101:11-6 doi: 10.1111/j.1651-2227.2011.02547.x.
20. Lund CH, Osborne JW. Validity and reliability of the neonatal skin condition score. *J Obstet Gynecol Neonatal Nurs* 2004;33:320-7.
21. Medicines and Healthcare products Regulatory Agency. Chlorhexidine solutions: reminder of the risk of chemical burns in premature infants. *Drug Saf Update* 2014;8:S3.
22. Maki DG, Weise CE, Sarafin HW. A semiquantitative culture method for identifying intravenous-catheter-related infection. *N Engl J Med* 1977;296:1305-9 doi: 10.1056/NEJM197706092962301.



For peer review only - http://bmjopen.bmj.com/site/about/guidelines.xhtml

| Procedure | Screening | Trial entry/ catheter insertion | Daily until 48 hours post-catheter removal | Catheter removal |
|---|---|---|---|---|
| Confirm Eligibility | √ | | | |
| Demographics | | √ | | |
| Consent | | √ | | |
| Randomisation | | √ | | |
| Open Study Pack/ catheter insertion | | √ | | |
| Skin Assessment [a] | | √ | √ | √ |
| Sepsis evaluation | | √ | √ | √ |
| Obtain blood culture if sepsis suspected [b] | | | √ | √ |
| Obtain two exit site skin swabs | | | | √ |
| Obtain proximal and tip catheter segments | | | | √ |
| SAE reporting [c] | | √ | √ | √ |
| Concomitant Medications (Antibiotics/antifungals only) | | √ | √ | √ |

a. Skin status to be recorded at baseline (prior to application of skin antiseptic), 10-30 minutes after catheter insertion, at 24 h (+/- 12 h), then daily until 48 h (+/- 12 h) after catheter removal using the modified neonatal contact dermatitis scoring system. Where catheter insertion is not achieved after IMP use, skin assessment for each anatomical region that was subject to IMP application will be recorded within 30 minutes following washing of the region with sterile water (to remove residual dried antiseptic compound), and then daily for a further 48 hours.

b. Peripheral blood culture obtained using standard aseptic technique.

c. Only adverse events which are serious will be recorded from the time of catheter insertion until 48 hours after catheter removal (or until 48 hours after antiseptic removal where catheterisation is unsuccessful). Only unforeseeable SAEs will be reported. Note that skin reactions to IMP use classed as moderate or severe should also to be notified to MHRA via the yellow card scheme in line with the specific current MHRA request for clinicians to report chlorhexidine-related skin burns in neonates.

Figure 1: Trial procedures

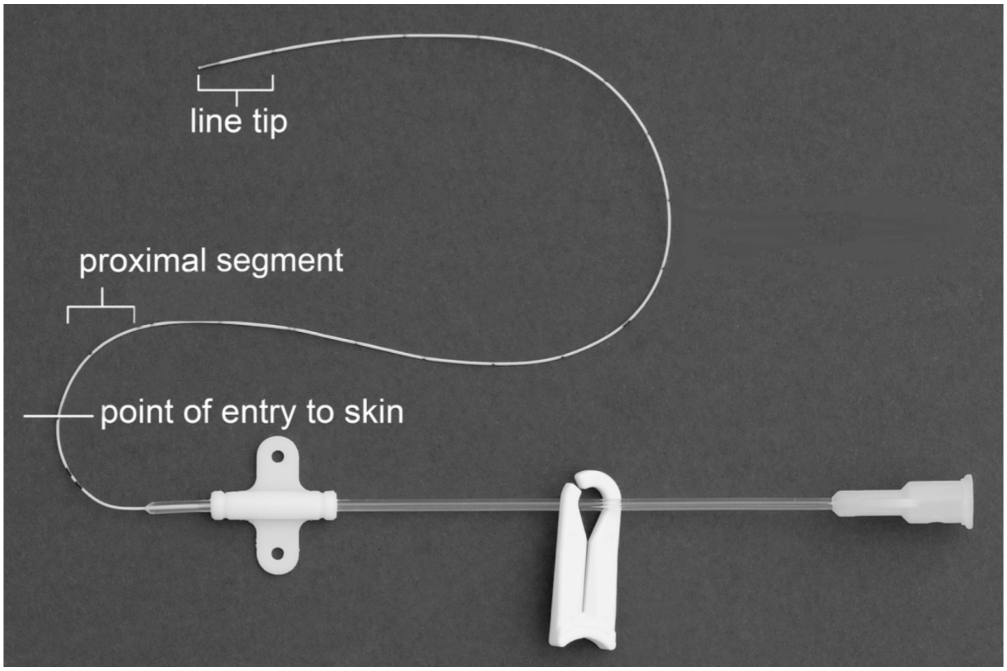


Figure 2: Catheter sections taken at the time of catheter removal

*(to be presented on NHS Trust headed paper)*

ARCTIC
Antiseptic Randomised Controlled Trial
for Insertion of Catheters

## INFORMATION FOR PARENTS ABOUT A RESEARCH STUDY

# Antiseptic Randomised Controlled Trial for Insertion of Catheters – The ARCTIC Study –

For peer review only

Dear Parents,

We would like to invite you to have your baby take part in a research study that is comparing two different skin antiseptics for cleaning the skin of babies. This leaflet explains the purpose of our study and what would be involved if you choose to allow your baby to take part.

**Why am I being asked about this study now?**
We are approaching you now because your baby will soon need to have a special feeding line inserted as part of their routine treatment on our neonatal unit. The special feeding line is a central venous catheter (CVC). This CVC is often also referred to as a 'long line' because it is a long, very thin silicone tube that is placed into one of your baby's arm/leg veins like a drip. It is 'long' because we aim to place the end of it in one of the big veins that drain into the heart. The CVC is needed to provide liquid nutrition that will help your baby to grow properly and become stronger. The CVC will stay in place until your baby manages to take all the milk needed for growth by mouth or feeding tube. These CVCs are usually required for at least 1-2 weeks, though sometimes for longer.

The study we are doing is looking at what antiseptic may be better to use to clean a baby's skin at the time when a CVC is inserted. We would therefore like you to consider allowing your baby to join this study before they have their CVC inserted in the next couple of days.

**Are any problems possible with central venous catheters?**
Insertion of CVCs into babies born prematurely is a routine procedure in neonatal units across the world. While these catheters are essential for delivering liquid nutrition and medicines to babies, one problem with their use is that they can sometimes attract bacteria and so lead to a bloodstream infection. Infection can be dangerous for small babies and so if there are concerns about possible infection in a baby who has a CVC in place, we would usually remove the catheter and treat the baby with antibiotics.

**How can catheter infection be prevented?**
A lot of research has already been done to understand and prevent catheter infections. Various good practices have been identified for insertion and management of CVCs, and you may be reassured to hear that our unit has adopted the good practices.


For peer review only - http://bmjopen.bmj.com/site/about/guidelines.xhtml

**Why is this study being done?**

One important aspect of catheter care to minimise infection is good skin disinfection at the time a CVC is inserted. Skin germs are the commonest cause of bloodstream infections in premature babies so choosing an effective skin antiseptic is clearly very important. Yet little research has been done in this area in babies, so we are trying to find out what antiseptics are best to use for CVC insertion in premature babies. Good antiseptic skin preparation before CVC insertion can significantly reduce the number of germs present on the skin and so reduce the risk of the skin germs colonising (growing onto) the catheter and leading to catheter infection. While it is standard practice to use an antiseptic solution to clean the skin at the time a CVC is inserted, at the moment we do not know which is the best antiseptic to use for babies. Currently in the UK many different antiseptics are being used in neonatal units and this is because we simply don't yet know which one works best or is safest to use for premature babies.

Our study is therefore comparing two commonly-used antiseptics for CVC insertion in premature babies. This is a small initial study (called a 'feasibility study') that will help us get some vital information about the use of these antiseptics in premature babies that will help us properly design a larger future study.

**Does my baby have to take part?**

It is entirely up to you whether you wish your baby to take part in this study. If you don't want your baby to take part, you do not have to give any reasons and neither your care nor your baby's will be affected. If you do take part, then you are still free to withdraw your baby from the study at any time, without giving any reason and without on-going care being affected.

**What will happen if my baby joins the study?**

If you consent to your baby taking part in this study, your baby will be randomly assigned to have their skin cleaned with either an alcohol-based antiseptic (70% isopropyl alcohol and 2% chlorhexidine) or a water-based antiseptic (2% chlorhexidine aqueous). The antiseptic used in your baby will be selected randomly by a special computer programme and will be used to disinfect your baby's skin both when their CVC is inserted and when it is removed. Neither you nor the staff caring for your baby will know which antiseptic your baby has been allocated.

After the CVC has been inserted, your baby will remain in the study until 2 days after its removal. During the study we will check daily for any skin reactions to the antiseptic. Also as part of this study, when the CVC gets removed we will take two skin swabs from the CVC insertion site to check for any bacteria and we will also send two small portions of the CVC itself away to the microbiology laboratory to check how many bacteria may have collected onto it. This will give us some initial information about how effective these two antiseptics may be at preventing CVC colonisation by skin bacteria.

Apart from the antiseptic used to clean your baby's skin and the skin swabs and catheter segments taken at CVC removal, all other aspects of the CVC insertion and removal will be completely in line with standard clinical management. No extra blood tests are required for this study. Being in the study will not interfere in any other way with the medical or nursing care your baby receives.

Catheters are usually removed because they are no longer needed (i.e. when a baby has reached full milk feeds and no longer needs direct liquid feeding into their bloodstream). But in about a quarter of babies who have a CVC in place, some signs of possible infection may develop before the CVC is due to be removed. If this happened to your baby while taking part in this study we would follow standard clinical practices, namely we would usually aim to remove the CVC early, send some routine blood tests to check for infection, and start your baby on antibiotics. But in addition, we will still send the study skin swabs and catheter


For peer review only - http://bmjopen.bmj.com/site/about/guidelines.xhtml

segments at CVC removal, because these may provide important additional information that will help us to know definitively whether your baby had developed a catheter-related infection. They will also allow us to check that any antibiotics your baby got started on were the right ones needed.

This study has been designed so that there is more chance of a baby receiving the alcohol-based antiseptic (3:1 ratio). The alcohol-based version is the commonest antiseptic being used in babies in the UK at present. Although we are interested to see how many catheters get colonised with bacteria with use of each of the two antiseptics being studied, the results from the alcohol-based antiseptic group will in particular help us know how many babies are needed for the large future study. This is why for this initial study any baby who participates is, on average, more likely to be allocated to the alcohol-based antiseptic. At the moment we don't know whether alcohol is an important component of the antiseptic in babies. The large future study will answer this question.

**What happens to the study samples?**
The skin swabs and catheter segments will be sent to the local microbiology laboratory of this hospital for initial testing. Any baby who has a blood sample taken for suspected infection during the study would also have this sample tested in this local microbiology laboratory, as per usual clinical practice. If any of these samples turn out to be positive on testing, i.e. show growth of bacteria, then a specimen of the bacteria grown (called an 'isolate') will be stored for more detailed testing at a later date. The further testing will be done after sending the isolates away to a specialist microbiology laboratory at the University of East Anglia where the exact species of bacteria will be identified with the help of advanced tests and equipment. All isolates sent away to the University laboratory will be fully anonymised, and identifiable only through linking to our database by the study number. Positive isolates will be kept for a period of 2 years after completion of the study before being destroyed.

**Are there any possible risks or side effects of taking part?**
Both antiseptic solutions chosen for use in this study are already commonly used in premature babies in Europe and America. Skin reactions such as redness and chemical burns have occasionally been reported with both of these antiseptics. The risk of skin reactions is increased when excess antiseptic solution is used or when it is in prolonged contact with the skin in very premature babies. Our study aims to minimise the risk of any reactions by carefully limiting the use of antiseptic to the minimum needed to cover the skin and by avoiding any prolonged contact of antiseptic with the baby's skin. We will check the skin closely for any reactions.

Skin reactions resulting from these two antiseptics appear to be uncommon in premature babies: one recent study from Ireland reported skin reactions in only 3 (2%) out of 148 premature babies treated with alcohol-based 2% chlorhexidine before CVC insertion; another study from Canada showed no adverse skin effects in any of the 199 premature babies whose skin was cleaned with the very same two skin antiseptics that we are now studying.

**Are there any potential benefits to my baby from taking part?**
We do not know if your baby will benefit directly from taking part in this study. If the antiseptic they receive in this study is more effective than the one normally used for CVC insertion, then they may benefit from a decreased risk of catheter infection. However this study is not designed to prove such possible benefit. The information we get from this initial study will nevertheless be important because it will help us to design a bigger study in the future. The future big study will test these same two antiseptics in a large number of babies to find out which one may be better. Doctors and nurses will then have a much better idea which antiseptic they should choose for skin preparation before CVC insertion in premature


For peer review only - http://bmjopen.bmj.com/site/about/guidelines.xhtml

babies to reduce the risk of catheter infection. We believe that the information from this initial study may therefore help towards improving the future care of premature babies.

**Will taking part in this study be kept confidential?**
Yes. At the beginning of the study your baby will be given a study number. This number (and not their name) will be used when studying and analysing the data. Information collected on your baby for this study will be entered electronically into a linked anonymised, password-protected, computer database, accessible by the researchers only. Your baby's involvement in this study will be noted in his/her medical records. With your permission we will let your GP know that your baby has taken part in this study. This will be done by including brief details about the study on the clinical summary that gets sent to your GP when your baby is discharged home from the neonatal unit.

With your consent we will collect some personal data to enable us to contact you in the future with the results of the research. Personal details will be kept for a period of no less than 10 years and will be kept only by the study organisers based in Norwich. At all times personal data will be held securely and will not be used for any other purpose. All other information collected for this study may also be used to help in future research studies but will never identify you or your baby. At all times the details will be handled only by authorised individuals and will remain confidential. Clinicians and research staff directly involved in the study will have direct access to your baby's medical records. In addition, employees of the Sponsor and/or the hospital Research and Development department and representatives from the Medicines and Healthcare products Regulatory Agency (MHRA) may require access to your baby's records as part of the monitoring procedures that are in place to oversee the conduct of the study. The full research records for this study will be retained for a period of not less than 25 years.

**What will happen to the results of the research?**
The results of this study will be shared with other doctors and nurses around the world who look after premature babies, so that they will be able to learn from the new information that might help them to improve their practice and care for babies. To do this a report containing the results of this study will be written, presented at scientific meetings and published in medical journals. Your baby's identity will not be made known in any circumstances.

**Who has reviewed this study?**
Before any research can go ahead in the NHS it needs to be checked by an independent group of people called a Research Ethics Committee. Their job is to ensure that any proposed research is ethical and to protect the safety, rights, well-being and dignity of participants. This study has been reviewed and approved by East of England – Cambridge South Research Ethics Committee, and also by the Research and Development department of this hospital. The study was also reviewed by the Neonatal Clinical Studies Group of the UK Medicines for Children Research Network (MCRN), and by Bliss (the premature baby charity). Some parents of premature babies that we previously cared for who required a CVC also helped us to design the study.

**Insurance and remuneration**
Your baby is covered by NHS insurance for any problems that may arise due to the study. If you consent for your baby to take part in this study you will not receive any payment or remuneration for their participation.

**What if new information becomes available during the study?**
We will discuss with you any important new information that may become available during the study which could affect your decision to let your baby take part.


For peer review only - http://bmjopen.bmj.com/site/about/guidelines.xhtml

**Who is organising and funding the study?**
The study is sponsored and by Norfolk and Norwich University Hospitals NHS Foundation Trust. The study has been funded by the National Institute for Health Research (NIHR) Research for Patient Benefit Programme.

**What if I have any further questions or concerns?**
If you have any questions or would like further information please ask one of the doctors or nurses caring for your baby, or alternatively please contact the doctor leading this study at this hospital directly:

| Local Principal Investigator: *(add contact details)* |
|---|

If you have any concerns about this study or the way it has been carried out, you should first contact your local study doctor named above who will do their best to address your queries. If you remain unhappy, you may contact your local Patient Advice and Liaison Service (PALS) for advice:

| PALS: *(add contact details)* |
|---|

**What do I do now?**
If you agree to your baby participating in this study, and have had all your questions answered satisfactorily, please complete and sign the consent form on the next page. You will be given a copy of the signed form to keep, along with this information sheet.

*Thank you for reading this leaflet*
*and for thinking about taking part in this study*



For peer review only - http://bmjopen.bmj.com/site/about/guidelines.xhtml

For peer review only

ARCTIC
Antiseptic Randomised Controlled Trial for Insertion of Catheters

# Maternal Consent Form

Please complete in black ballpoint pen

**Hospital name:** ________________________ **Study Number:** [ ][ ][ ]

| Baby's first name | Baby's last name |
|---|---|
| | |

**Title of study:** ARCTIC Trial
**Formal Title:** Antiseptic Randomised Controlled Trial for Insertion of Catheters
**Chief Investigator:** Dr Paul Clarke

**Please initial box**

1. I confirm that I have read and understood the information in the Parental Informed Consent Form for this study (v 1.2, 18th November 2016) and have had the opportunity to consider the information, ask questions, and have had these answered satisfactorily. [ ]
2. I understand that participation in this study is voluntary and that I am free to withdraw my baby from the study at any time without giving any reason, and without my or my baby's present or future medical care or legal rights being affected. [ ]
3. I understand that relevant sections of medical notes and data collected during the study relating to me and my baby may be looked at by the researchers and by the Sponsor, Funder, regulatory authorities or my NHS Trust. I give permission for these individuals to have access to these notes where it is relevant to taking part in this research. [ ]
4. I agree that my personal contact details can be collected, stored, and sent to the co-ordinating centre in Norwich, along with a copy of this signed consent form, to enable follow-up contact with me regarding this study. This is on the understanding that any such information will be treated confidentially. [ ]
5. I understand that information held and managed by the Health and Social Care Information Centre and other central UK NHS bodies may be used to help contact me in the future or to provide information about my baby's health status. I also agree that data collected in this study may be used to help in future research studies. [ ]
6. I agree to my GP being informed of my baby's participation in the study. [ ]
7. I agree that my baby may take part in the ARCTIC study. [ ]

| Mother's Name | Name of health professional taking consent |
|---|---|
| Mother's Signature | Signature |
| D D / M M / Y Y | D D / M M / Y Y |

EudraCT No. 2015-000874-36. REC Ref. 15/EE/0345
Protocol Version 3.0, 18th November 2016

This project was funded by the National Institute for Health Research Research for Patient Benefit (RfPB) Programme (Project reference: PB-PG-1013-32076)

NPEU Clinical Trials Unit, Nuffield Department of Population Health
University of Oxford, Old Road Campus, Headington, Oxford, OX3 7LF
T: 01865 289700 F: 01865 289701
E: arctic@npeu.ox.ac.uk www.npeu.ox.ac.uk/arctic

UNIVERSITY OF OXFORD
npeu Clinical Trials Unit

For peer review only - http://bmjopen.bmj.com/site/about/guidelines.xhtml

# Reporting checklist for protocol of a clinical trial.

Based on the SPIRIT guidelines.

## Instructions to authors

Complete this checklist by entering the page numbers from your manuscript where readers will find each of the items listed below.

Your article may not currently address all the items on the checklist. Please modify your text to include the missing information. If you are certain that an item does not apply, please write "n/a" and provide a short explanation.

Upload your completed checklist as an extra file when you submit to a journal.

In your methods section, say that you used the SPIRIT reporting guidelines, and cite them as:

Chan A-W, Tetzlaff JM, Altman DG, Laupacis A, Gøtzsche PC, Krleža-Jerić K, Hróbjartsson A, Mann H, Dickersin K, Berlin J, Doré C, Parulekar W, Summerskill W, Groves T, Schulz K, Sox H, Rockhold FW, Rennie D, Moher D. SPIRIT 2013 Statement: Defining standard protocol items for clinical trials. Ann Intern Med. 2013;158(3):200-207

| | | Reporting Item | Page Number |
|---|---|---|---|
| Title | #1 | Descriptive title identifying the study design, population, interventions, and, if applicable, trial acronym | **1** |
| Trial registration | #2a | Trial identifier and registry name. If not yet registered, name of intended registry | **4** |
| Trial registration: data set | #2b | All items from the World Health Organization Trial Registration Data Set | **N/A - included in ISRCTN Trial Registration, not in this manuscript.** |
| Protocol version | #3 | Date and version identifier | **21** |
| Funding | #4 | Sources and types of financial, material, and other support | **24** |
| Roles and responsibilities: contributorship | #5a | Names, affiliations, and roles of protocol contributors | **1-2, & 24** |

For peer review only

| | | | |
|---|---|---|---|
| Roles and responsibilities: sponsor contact information | #5b | Name and contact information for the trial sponsor | 25 |
| Roles and responsibilities: sponsor and funder | #5c | Role of study sponsor and funders, if any, in study design; collection, management, analysis, and interpretation of data; writing of the report; and the decision to submit the report for publication, including whether they will have ultimate authority over any of these activities | 25 |
| Roles and responsibilities: committees | #5d | Composition, roles, and responsibilities of the coordinating centre, steering committee, endpoint adjudication committee, data management team, and other individuals or groups overseeing the trial, if applicable (see Item 21a for data monitoring committee) | 21 |
| Background and rationale | #6a | Description of research question and justification for undertaking the trial, including summary of relevant studies (published and unpublished) examining benefits and harms for each intervention | 6-8 |
| Background and rationale: choice of comparators | #6b | Explanation for choice of comparators | 7-8 |
| Objectives | #7 | Specific objectives or hypotheses | 8 |
| Trial design | #8 | Description of trial design including type of trial (eg, parallel group, crossover, factorial, single group), allocation ratio, and framework (eg, superiority, equivalence, non-inferiority, exploratory) | 9, 11 |
| Study setting | #9 | Description of study settings (eg, community clinic, academic hospital) and list of countries where data will be collected. Reference to where list of study sites can be obtained | 9 |

For peer review only

| | | | |
|---|---|---|---|
| Eligibility criteria | #10 | Inclusion and exclusion criteria for participants. If applicable, eligibility criteria for study centres and individuals who will perform the interventions (eg, surgeons, psychotherapists) | **9, 10** |
| Interventions: description | #11a | Interventions for each group with sufficient detail to allow replication, including how and when they will be administered | **11-14** |
| Interventions: modifications | #11b | Criteria for discontinuing or modifying allocated interventions for a given trial participant (eg, drug dose change in response to harms, participant request, or improving / worsening disease) | **16** |
| Interventions: adherance | #11c | Strategies to improve adherence to intervention protocols, and any procedures for monitoring adherence (eg, drug tablet return; laboratory tests) | **N/A - this is feasibility trial and adherence is one of outcomes being assessed.** |
| Interventions: concomitant care | #11d | Relevant concomitant care and interventions that are permitted or prohibited during the trial | **15** |
| Outcomes | #12 | Primary, secondary, and other outcomes, including the specific measurement variable (eg, systolic blood pressure), analysis metric (eg, change from baseline, final value, time to event), method of aggregation (eg, median, proportion), and time point for each outcome. Explanation of the clinical relevance of chosen efficacy and harm outcomes is strongly recommended | **15-16** |
| Participant timeline | #13 | Time schedule of enrolment, interventions (including any run-ins and washouts), assessments, and visits for participants. A schematic diagram is highly recommended (see Figure) | **11, Figure 1** |
| Sample size | #14 | Estimated number of participants needed to achieve study objectives and how it was determined, including clinical and statistical | **17-18** |

For peer review only

| | | | |
|---|---|---|---|
| | | assumptions supporting any sample size calculations | |
| Recruitment | #15 | Strategies for achieving adequate participant enrolment to reach target sample size | 10, 11, 17-18 |
| Allocation: sequence generation | #16a | Method of generating the allocation sequence (eg, computer-generated random numbers), and list of any factors for stratification. To reduce predictability of a random sequence, details of any planned restriction (eg, blocking) should be provided in a separate document that is unavailable to those who enrol participants or assign interventions | 11 |
| Allocation concealment mechanism | #16b | Mechanism of implementing the allocation sequence (eg, central telephone; sequentially numbered, opaque, sealed envelopes), describing any steps to conceal the sequence until interventions are assigned | 11 |
| Allocation: implementation | #16c | Who will generate the allocation sequence, who will enrol participants, and who will assign participants to interventions | 10, 11 |
| Blinding (masking) | #17a | Who will be blinded after assignment to interventions (eg, trial participants, care providers, outcome assessors, data analysts), and how | 12, 16-17 |
| Blinding (masking): emergency unblinding | #17b | If blinded, circumstances under which unblinding is permissible, and procedure for revealing a participant's allocated intervention during the trial | 16-17 |
| Data collection plan | #18a | Plans for assessment and collection of outcome, baseline, and other trial data, including any related processes to promote data quality (eg, duplicate measurements, training of assessors) and a description of study instruments (eg, questionnaires, laboratory tests) along with their reliability and validity, if | 18-19 |

For peer review only

| | | | |
|---|---|---|---|
| | | known. Reference to where data collection forms can be found, if not in the protocol | |
| Data collection plan: retention | #18b | Plans to promote participant retention and complete follow-up, including list of any outcome data to be collected for participants who discontinue or deviate from intervention protocols | **N/A - retention rates is one of the secondary outcome measures which this feasibility study is assessing** |
| Data management | #19 | Plans for data entry, coding, security, and storage, including any related processes to promote data quality (eg, double data entry; range checks for data values). Reference to where details of data management procedures can be found, if not in the protocol | **18-19** |
| Statistics: outcomes | #20a | Statistical methods for analysing primary and secondary outcomes. Reference to where other details of the statistical analysis plan can be found, if not in the protocol | **16-18** |
| Statistics: additional analyses | #20b | Methods for any additional analyses (eg, subgroup and adjusted analyses) | **19** |
| Statistics: analysis population and missing data | #20c | Definition of analysis population relating to protocol non-adherence (eg, as randomised analysis), and any statistical methods to handle missing data (eg, multiple imputation) | **N/A - this is feasibility study and rates of non completion/missing primary outcome data will be reported as outcomes.** |
| Data monitoring: formal committee | #21a | Composition of data monitoring committee (DMC); summary of its role and reporting structure; statement of whether it is independent from the sponsor and competing interests; and reference to where further details about its charter can be found, if not in the protocol. Alternatively, an explanation of why a DMC is not needed | **21** |
| Data monitoring: interim analysis | #21b | Description of any interim analyses and stopping guidelines, including who will have | **21** |

For peer review only

| | | | |
|---|---|---|---|
| | | access to these interim results and make the final decision to terminate the trial | |
| Harms | #22 | Plans for collecting, assessing, reporting, and managing solicited and spontaneously reported adverse events and other unintended effects of trial interventions or trial conduct | **20-21** |
| Auditing | #23 | Frequency and procedures for auditing trial conduct, if any, and whether the process will be independent from investigators and the sponsor | **20** |
| Research ethics approval | #24 | Plans for seeking research ethics committee / institutional review board (REC / IRB) approval | **21-22** |
| Protocol amendments | #25 | Plans for communicating important protocol modifications (eg, changes to eligibility criteria, outcomes, analyses) to relevant parties (eg, investigators, REC / IRBs, trial participants, trial registries, journals, regulators) | **N/A - there were no further changes to the protocol v3.0 dated 18 NOV 2016 during the active recruitment phase.** |
| Consent or assent | #26a | Who will obtain informed consent or assent from potential trial participants or authorised surrogates, and how (see Item 32) | **10** |
| Consent or assent: ancillary studies | #26b | Additional consent provisions for collection and use of participant data and biological specimens in ancillary studies, if applicable | **N/A** |
| Confidentiality | #27 | How personal information about potential and enrolled participants will be collected, shared, and maintained in order to protect confidentiality before, during, and after the trial | **18-19** |
| Declaration of interests | #28 | Financial and other competing interests for principal investigators for the overall trial and each study site | **25** |
| Data access | #29 | Statement of who will have access to the final trial dataset, and disclosure of contractual agreements that limit such access for investigators | **25** |

For peer review only

| | | | |
|---|---|---|---|
| Ancillary and post trial care | #30 | Provisions, if any, for ancillary and post-trial care, and for compensation to those who suffer harm from trial participation | **N/A** |
| Dissemination policy: trial results | #31a | Plans for investigators and sponsor to communicate trial results to participants, healthcare professionals, the public, and other relevant groups (eg, via publication, reporting in results databases, or other data sharing arrangements), including any publication restrictions | **21-22** |
| Dissemination policy: authorship | #31b | Authorship eligibility guidelines and any intended use of professional writers | **N/A - eligibility for authorship will follow ICMJE guidelines.** |
| Dissemination policy: reproducible research | #31c | Plans, if any, for granting public access to the full protocol, participant-level dataset, and statistical code | **21** |
| Informed consent materials | #32 | Model consent form and other related documentation given to participants and authorised surrogates | **Supplementary Appendix 1** |
| Biological specimens | #33 | Plans for collection, laboratory evaluation, and storage of biological specimens for genetic or molecular analysis in the current trial and for future use in ancillary studies, if applicable | **14-15** |

The SPIRIT checklist is distributed under the terms of the Creative Commons Attribution License CC-BY-ND 3.0. This checklist can be completed online using https://www.goodreports.org/, a tool made by the EQUATOR Network in collaboration with Penelope.ai

For peer review only
