# Supplementary material for: Safety and efficacy of 2% chlorhexidine gluconate aqueous versus 2% chlorhexidine gluconate in 70% isopropyl alcohol for skin disinfection prior to percutaneous central venous catheter insertion in preterm neonates: the ARCTIC randomised-controlled feasibility trial protocol
Source: BMJ Open. 2019 Feb 8;9(2):e028022. doi: 10.1136/bmjopen-2018-028022 (PMC6411256; doi:10.1136/bmjopen-2018-028022)
Supplement: Reviewer comments [file bmjopen-2018-028022.reviewer_comments.pdf]

PEER REVIEW HISTORY

BMJ Open publishes all reviews undertaken for accepted manuscripts. Reviewers are asked to complete a checklist review form (**http://bmjopen.bmj.com/site/about/resources/checklist.pdf**) and are provided with free text boxes to elaborate on their assessment. These free text comments are reproduced below.

ARTICLE DETAILS

**TITLE (PROVISIONAL)**	Safety and efficacy of 2% chlorhexidine gluconate aqueous versus 2% chlorhexidine gluconate in 70% isopropyl alcohol for skin disinfection prior to percutaneous central venous catheter insertion in preterm neonates: the ARCTIC randomised-controlled feasibility trial protocol
**AUTHORS**	Clarke, Paul; Craig, Jean V; Wain, John; Tremlett, Catherine; Linsell, Louise; Bowler, Ursula; Juszczak, Ed; Heath, Paul T

This article was not externally reviewed at BMJ Open. Protocols that have been independently assessed prior to submission to BMJ Open are usually fast-tracked to publication on the grounds that further substantial changes will not be possible. This independent assessment will usually be external, independent review for both a substantial grant award from a non-commercial or government funder and ethics approval.
